# The Effect of the *N*-Oxide Oxygen Atom on the Crystalline and Photophysical Properties of [1,2,5]Oxadiazolo[3,4-*d*]pyridazines

**DOI:** 10.3390/molecules30112374

**Published:** 2025-05-29

**Authors:** Timofey N. Chmovzh, Alisia V. Tsorieva, Vladislav M. Korshunov, Egor D. Kotov, Darina I. Nasyrova, Mikhail E. Minyaev, Nikolay P. Datskevich, Ilya V. Taydakov, Michail N. Elinson, Oleg A. Rakitin

**Affiliations:** 1 N. D. Zelinsky Institute of Organic Chemistry, Russian Academy of Sciences, 119991 Moscow, Russia; tim1661@yandex.ru (T.N.C.); egor.kotov.dm@gmail.com (E.D.K.); nasyrova-01@mail.ru (D.I.N.); mminyaev@ioc.ac.ru (M.E.M.); 2 D. I. Mendeleev University of Chemistry and Technology of Russia, Miusskaya sqr., 9, 125047 Moscow, Russia; 3 P. N. Lebedev Physical Institute, Russian Academy of Sciences, 119991 Moscow, Russia; tsorievaav@gmail.com (A.V.T.); vladkorshunov@bk.ru (V.M.K.); dac1@yandex.ru (N.P.D.); taidakov@gmail.com (I.V.T.); 4 Skoltech Center for Energy Science and Technology, Skolkovo Institute of Science and Technology, 121205 Moscow, Russia

**Keywords:** [1,2,5]oxadiazolo[3,4-*d*]pyridazine, [1,2,5]oxadiazolo[3,4-*d*]pyridazine 1-oxide, donor-acceptor-donor molecule, luminescence, chalcogen, quantum yield, X-ray analysis

## Abstract

A series of novel fluorescent donor–acceptor–donor (D-A-D) dyes containing [1,2,5]oxadiazolo[3,4-*d*]pyridazine and its 1-oxide as electron-withdrawing groups has been synthesized and thoroughly investigated using X-ray diffraction and molecular spectroscopy methods. This study showed that the introduction of *N*-oxide into the 1,2,5-oxadiazole ring in the acceptor fragment leads to a significant decrease in the luminescence intensity and quantum yield of the dyes. A comprehensive comparison of the photophysical properties of the obtained compounds containing the 1,2,5-oxadiazole ring with the previously studied [1,2,5]thia- and 1,2,5-selenadiazolo[3,4-*d*]pyridazine analogs showed that the oxygen substitution in the acceptor fragment shifts the phosphorescence maximum from the NIR region of 980–1100 nm to the red region of 690–770 nm. In contrast, for oxygen- and sulfur-containing dyes, purely red fluorescence with a maximum in the spectral range of 620–900 nm is observed. The crystal structures of furoxan-containing **3d**·½CHCl_3_ and furazan-containing **4d** exhibit a non-planar [1,2,5]oxadiazolo[3,4-*d*]pyridazine fragment. We have found that short non-covalent interactions of the furoxan system with a lattice chloroform molecule in **3d** lead to luminescence quenching. Meanwhile, in the **4d** dye, the intermolecular π-π interactions of pyridazine nitrogen atoms with the *N*-carbazolyl group of the adjacent molecule should facilitate intermolecular charge transfer (ICT) emission. Thus, the luminescence maxima for these dyes can be tuned across a broad range of 700–1100 nm by varying the number of chalcogen atoms, highlighting the potential for tailoring optical properties in optoelectronic applications.

## 1. Introduction

Over the past two decades, donor–acceptor (D-A) molecules based on 1,2,5-chalcogenadiazoles have aroused significant scientific interest due to their potential applications in various optoelectronic devices. These molecules are of particular interest due to their unique physical properties, such as visible light absorption, extended π-conjugation that enhances molar extinction coefficients, and efficient charge carrier delocalization, which are essential for the performance of devices such as Grätzel cells. In addition, the applications include bulk heterojunction [1,2] dye-sensitized solar cells [3,4], organic light-emitting diodes [5,6], and n-type field-effect transistors [7,8].

Photovoltaic applications rely on light absorption, whereas electroluminescent devices require controlled emission within specific spectral ranges. However, the chemical design of D-A dyes with specified luminescence properties remains challenging, primarily due to the lack of systematic approaches for predicting absorption and emission spectra prior to their synthesis. Recent efforts have focused on developing criteria to guide the design of such dyes. For instance, symmetric donor–acceptor–donor (D-A-D) structures have exhibited enhanced intramolecular charge transfer (ICT) in comparison to more simplistic D-A systems [9]. Through the modification of the donor and acceptor moieties, the energy gap between the highest occupied molecular orbital (HOMO) and the lowest unoccupied molecular orbital (LUMO) can be fine-tuned, thereby adjusting the absorption and emission maxima.

In our recent work, we demonstrated that D-A-D compounds incorporating 1,2,5-thiadiazolo[3,4-*d*]pyridazine as the central acceptor, with indoline and carbazole derivatives as donors, emit in the visible to near-infrared (NIR) range. These compounds were successfully employed as emitters in NIR OLEDs, achieving high external quantum efficiency [10]. Furthermore, the utilization of [1,2,5]selenadiazolo[3,4-*d*]pyridazine as the central acceptor resulted in dyes exhibiting dual emission spanning 700–900 nm and 900–1100 nm, thereby violating Kasha’s rule [11].

In our previous study, it was demonstrated that D-A-D-type molecules based on [1,2,5]oxadiazolo[3,4-*d*]pyridazine, with carbazole and cyclohexaindoline donor fragments, exhibited red emission [12]. However, the luminescent properties of [1,2,5]oxadiazolo[3,4-*d*]pyridazine 1-oxide derivatives remain unexplored, and a systematic comparison of the optical properties of chalcogen-based acceptors is still lacking.

**Figure 1:**
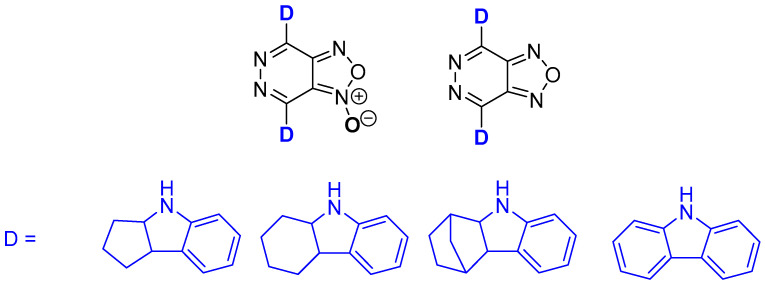
Chemical structures of the synthesized dyes.

This study represents the culmination of a comprehensive research cycle, with the objective of elucidating the influence of heavy atoms in the acceptor fragment of chalcogenadiazole-based D-A-D dyes on their luminescent properties. Specifically, we investigate how different acceptor fragments, combined with identical donor units, affect emission in the visible and NIR regions. We report the synthesis and characterization of a series of novel D-A-D compounds based on [1,2,5]oxadiazolo[3,4-*d*]pyridazine (furazan) and its 1-oxide (furoxan) paired with donors such as 1,2,3,3a,4,8b-hexahydrocyclopenta[*b*]indole, 1,2,3,3a,4,8b-hexahydrocyclohexa[*b*]indole, 1,2,3,4,4a,4a,9a-hexahydro-9λ^2^-1,4-methanocarbazole, and carbazole (Figure 1). A comparison of these results with data from previous studies [11,12] establishes a clear relationship between the chalcogen atom in the acceptor fragment and the emission spectral range, providing valuable insights for the design of optoelectronic materials.

## 2. Results and Discussion

### 2.1. Synthesis

**Scheme 1:**
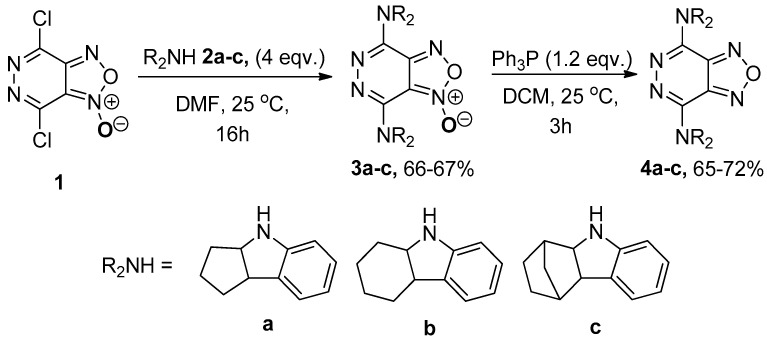
Synthesis of [1,2,5]oxadiazolo[3,4-*d*]pyridazines and [1,2,5]oxadiazolo[3,4-*d*]pyridazine 1-oxides.

We have studied the substitution reactions of chlorine atoms in the pyridazine ring by amino groups. It was demonstrated that when the reaction between dichloride **1** and amines **2**(**a**–**c**) (4 equiv.) was carried out in DMF at room temperature, the formation of bis-substitution products **3**(**a**–**c**) occurred in high yields. The reduction of the furoxan ring to the furazan cycle was carried out under the action of triphenylphosphine in dichloromethane at room temperature for 3 h to obtain the target dyes **4**(**a**–**c**), the yield of which varied within 65–72% (Scheme 1).

**Scheme 2:**
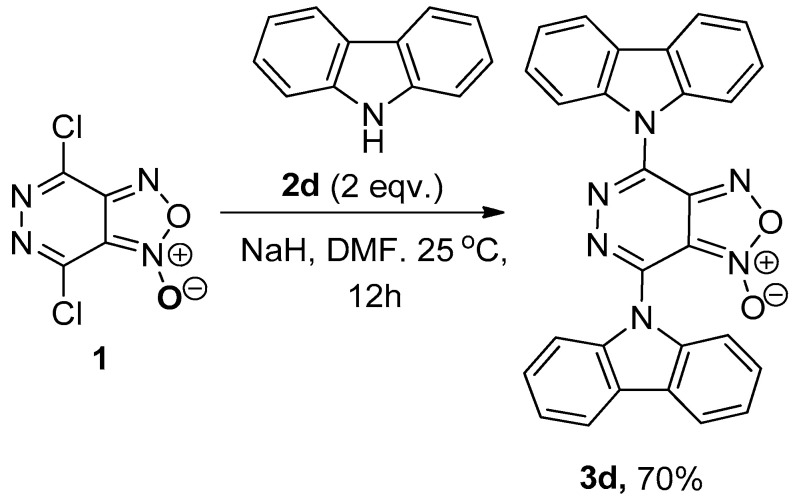
Synthesis of 4,7-di(9*H*-carbazol-9-yl)-[1,2,5]oxadiazolo[3,4-*d*]pyridazine 1-oxide **3d**.

**Figure 2:**
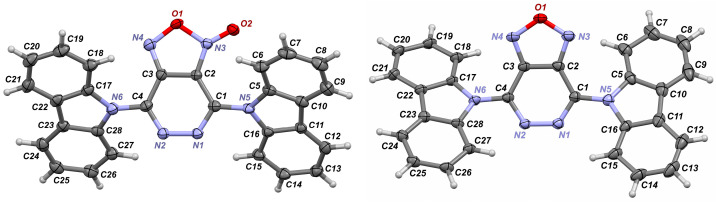
Crystal structures of **3d** ((**left**); only one crystallographicaly unique molecule is shown; the disorder of the furoxan cycle is omitted) and **4d** (**right**). Herein and thereafter, anisotropic displacement parameters are set to a 50% probability level.

It has been demonstrated that carbazole reacts with 4,7-dichloro[1,2,5]oxadiazolo[3,4-*d*]pyridazine 1-oxide **1**. It has been established that the use of the sodium salt of this amine in DMF for 24 h at 25 °C results in the formation of the bis-substitution product **3d** in moderate yield (Scheme 2). The structure of the **3d** compound was unambiguously confirmed via X-ray structural analysis (Figure 2).

**Scheme 3:**
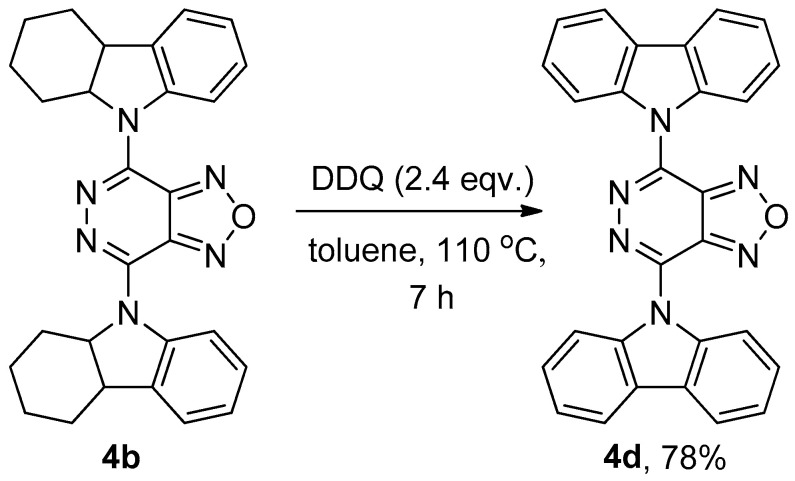
Synthesis of 4,7-di(9*H*-carbazol-9-yl)-[1,2,5]oxadiazolo[3,4-*d*]pyridazine (**4d**).

It was found that the **4d** compound could be successfully synthesized from bis(hexahydrocarbazolyl) derivative **4b** via dehydrogenation in toluene with 4,5-dichloro-3,6-dioxocyclohexa-1,4-diene-1,2-dicarbonitrile (DDQ) to form the target product **4d** with a high yield (Scheme 3) [13]. The structure of compound **4d** was unambiguously confirmed using X-ray crystallography (Figure 2).

### 2.2. X-Ray Analysis and DFT Calculations

The crystal structures of **3d**·½CHCl_3_ and **4d** have been studied using the single-crystal X-ray diffraction analysis. The asymmetric unit of **3d**·½CHCl_3_ contains a disordered lattice chloroform molecule and two symmetrically non-equivalent molecules of **3d** (Figure 2, left), whereby the furoxan fragment (C_2_N_2_O_2_) is disordered over two positions (see Supplementary Materials (ESI) for details). Both specimens crystalize in orthorhombic space groups (non-centrosymmetric *Pca2_1_*, Z′ = 2 for **3d**·½CHCl_3_ and centrosymmetric *Pbca*, Z′ = 1 for **4d**) with Z = 8 in both cases. The unit cell parameters and packing plots are similar but differ slightly; the presence of the solvent molecule and the disorder of the furoxan fragment lead to a loss of symmetry elements (two 2_1_ screw axes and a glide plane) in **3d**·½CHCl_3_.

The selected bond distances for **3d** and **4d** are provided in Table 1. Expectedly, the furoxan fragments reveals a significant elongation of the O1-N3/O3-N10 bond by 0.10 Å and an elongation of the N3-C2/N10-C31 bond by 0.02 Å located at the *N*-oxide fragment in **3d**, compared to those in the furazan fragment of **4d**. In order to compare geometrical parameters, DFT calculations were performed at the PBE0/DEF2TZVP level of theory in the gas phase at 298K. Calculated bond distances are provided in Table 2. They are in good or in fairly good agreement with crystallographic data.

Both molecules **3d** and **4d** demonstrate that the entire heterocycle N_4_C_4_O_2_ (**3d**)/N_4_C_4_O (**4d**) is not flat and demonstrates slight folding along two lines passing through the following atom pairs: C1/C4 and C2/C3. The folding angles (see ESI) are rather small (in the case of **3d**, we have only evaluated its values due to high ESDs caused by the disorder of the furoxan cycle), not preventing a good conjugation within the π-system of the heterocycle. We suppose that the non-planarity [1,2,5]oxadiazolo[3,4-*d*]pyridazine heterocycle is not flat, likely due to intrinsic electronic effects rather than intra- and intermolecular van der Waals interactions (see below). Our idea was successfully confirmed by results of quantum calculations (see dihedral angles for optimized structures, Tables S9 and S10 in Supplementary Information).

On the contrary, nearly flat *N*-carbazolyl groups (C_12_H_8_N) are rotated by 29.38(6)° and 40.02(5)° with respect to the N_2_C_4_ plane of the central heterocycle in **4d**, indicating that π-π conjugation between the C_12_H_8_N and N_4_C_4_O heterocycle systems is lost to a substantial extent. The presence of an additional oxygen atom in the furoxan ring makes the situation even more dramatic for **3d**: the values of C_12_H_8_N-N_2_C_4_ dihedral rotation angles lie in the range of 41.6(1.5) to 46.3(1)° (see ESI). Such high values of dihedral angles are clearly the result of intramolecular van der Waals interactions within the molecule. Consequently, the *N*-carbazolyl group is unable to function as an effective antenna to enhance the luminescent properties of the investigated dyes. The optimized geometry of **3d** perfectly reproduces C_12_H_8_N-N_2_C_4_ dihedral rotation angles. However, the optimized geometry of **4d** indicates that both C_12_H_8_N-N_2_C_4_ dihedral angles should be of 47° in a gas phase, which contradicts with observations of the crystal structure, where one angle is much smaller. The latter is clearly induced by non-covalent interactions in a crystalline phase.

**Figure 3:**
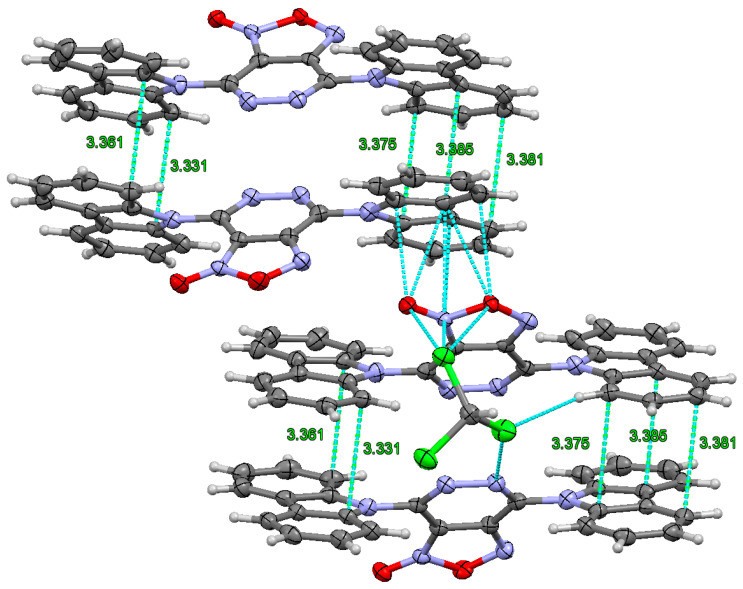
Intermolecular contacts of carbazole rings in the crystal of **3d**, forming π-π stacking, and short contacts of the furoxan ring.

In **3d**, pronounced π-π stacking is formed with an average distance of 3.35 Å between the π-systems (Figure 3). This phenomenon can be attributed to the minor deviation of the carbazolyl aromatic systems from each other within the crystal packing (Figure S3 in ESI). It should be noticed that the furoxan ring forms non-covalent contacts with a carbazolyl group of a neighboring molecule and with a lattice chloroform molecule. This observation potentially elucidates the comparatively inferior photophysical properties exhibited by **3d** in comparison to **4d**, as evidenced by phenomena such as luminescence quenching.

**Figure 4:**
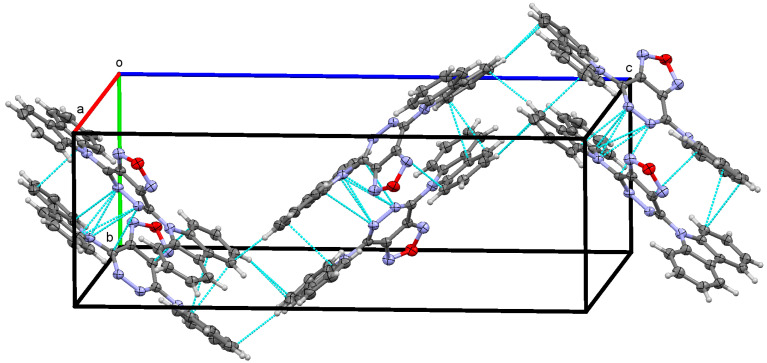
Zigzag-shaped layers in the crystal packing of **4d**. Only two layers are shown for clarity.

**Figure 5:**
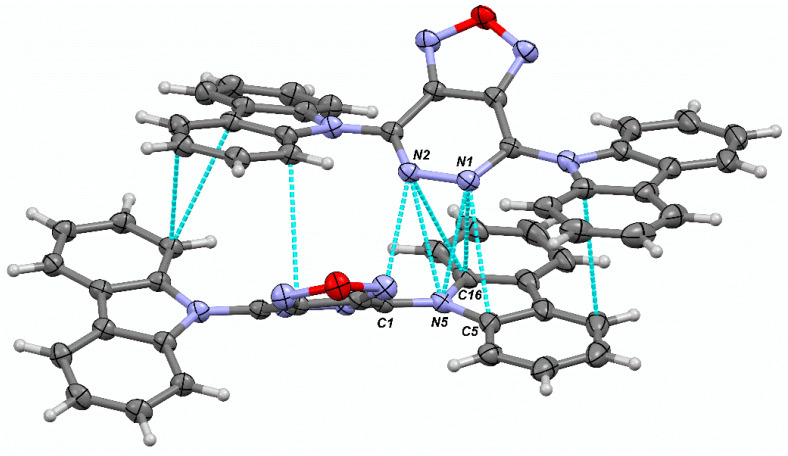
Intermolecular non-valent contacts between two adjacent molecules of **4d**.

In the crystal structure of **4d**, the molecules are arranged in zigzag-shaped layers, supported by intermolecular π-π interactions of aromatic rings, including short contacts between nitrogen atoms (Figure 4); the angle in the zigzag is close to 90°. The distances between *N*-carbazolyl systems of neighboring molecules are in the range of 3.31 Å to 3.37 Å. More pronounced non-covalent interactions are between atoms N1 and N2 of one molecule and the N5, C1, C5, and C16 atoms of the *N*-carbazolyl substituent of another molecule (namely, the group that exhibits a smaller rotation angle): 2.959 Å for N1···N5, 3.061 Å for N1···C16, 3.241 Å for N1···C5, 3.024 Å for N2···C1, 3.080 Å for N2···N5, and 3.248 Å for N2···C16 (Figure 5). These particular interactions hinder the rotation of one of the two *N*-carbazolyl fragments to a greater angle, arrange molecules of **4d** into 1D chains oriented along the *b* direction (see ESI), and should facilitate charge transfer from one molecule to another, enhancing its photophysical properties in the solid state, compared to those in **3d**.

Therefore, the crystal structure of the **3d** compound reveals not only π-π stacking but also short intermolecular interactions between the furoxan rings that lead to luminescence quenching.

On the contrary, π-π stacking in the **4d** compound containing the furazan system is observed primarily between two *N*-carbazolyl groups and between the pyridazine heterocycle and the *N*-carbazolyl group of adjacent molecules; the [1,2,5]oxadiazolo[3,4-*d*]pyridazine system might be considered as nearly unperturbed by intermolecular interactions. Consequently, the non-flat nature of the furazan system is likely caused by electronic effects.

### 2.3. Photophysical Properties

**Figure 6:**
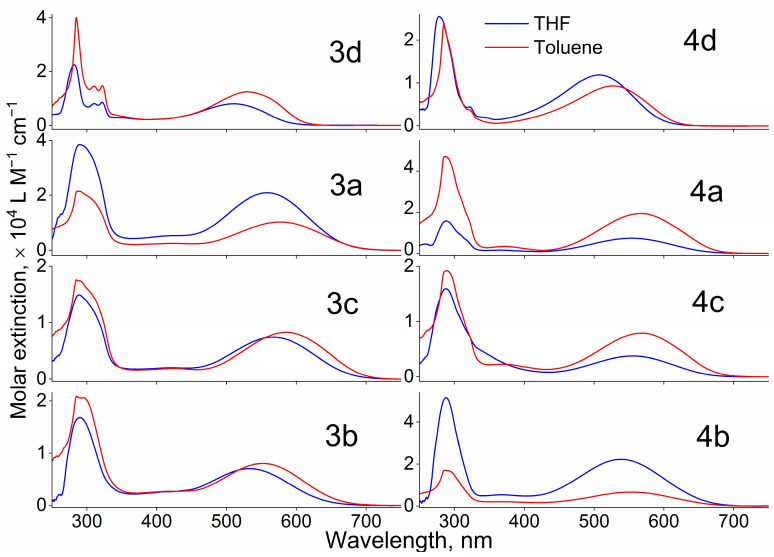
UV-Vis spectra recorded for all investigated dyes dissolved in THF and toluene.

Optical absorption spectra measured for all investigated compounds dissolved in THF and toluene with a concentration of about 10^−5^ M/L reveal several bands (see Figure 6). Since the bands with the maxima at 280–295 nm depend on the nature of the solvent insignificantly, they are characterized by π-π* transitions. The long-wavelength band blue-shifts with the increase in solvent polarity for all dyes, so it is associated with the ICT state. Due to donor fragment variations from carbazole (**3d** and **4d** dyes) to the **b**, **a**, and **c** moieties, the ICT peaks shift bathochromically by 25–50 nm. The photophysical parameters such as the absorption maximum wavelength (λ*_abs_*), maximum molar extinction for the ICT band (*ε*_max_), photoluminescence (PL) maximum wavelength (λ*_em_*), and Stokes shift (Δ*ν*) are listed in Table 3.

**Figure 7:**
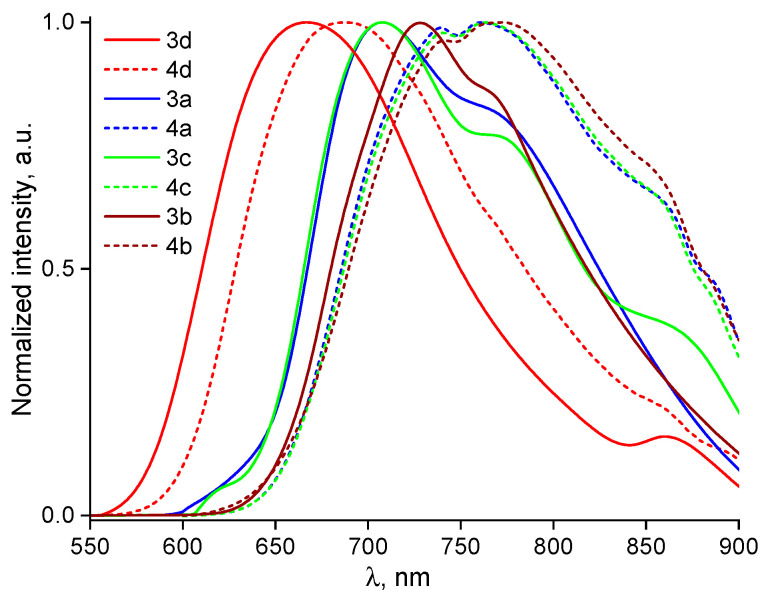
Normalized PL spectra recorded for all dyes dissolved in toluene upon photoexcitation at the λ*_abs_* of the ICT state.

Comparing compounds containing 1,2,5-oxadiazole (furazan) and 1,2,5-oxadiazole 2-oxide (furoxan) as acceptor fragments while using identical donor moieties, we observe qualitatively similar shapes in their absorption spectra. However, luminescence intensity strongly depends on the acceptor fragment. PL spectra were measured for all investigated dyes dissolved in toluene and THF upon excitation at λ*_abs_* corresponding to the ICT state (see Figure 7 and Figure S10). The dyes emit their spectra in the orange-red spectral region. The λ*_em_* of the **4d**, **4a**, **4c**, and **4b** compounds is red-shifted by 11–56 nm in comparison to analogs with 1,2,5-oxadiazole 2-oxide acceptor (**3d**, **3a**, **3c**, and **4b**). The PL spectra for the **3a**, **3c**, and **3b** compounds dissolved in toluene demonstrate a low signal-to-noise ratio that can be associated with more efficient non-radiative relaxation. The dominant non-radiative relaxation pathway can be explained by OH bonding of the oxygen at the N(1) position of the furoxan ring and hydrogen at the donor fragment. The emission spectra of compounds 3a and 3b in THF could not be accurately recorded due to their low luminescence intensity.

**Figure 8:**
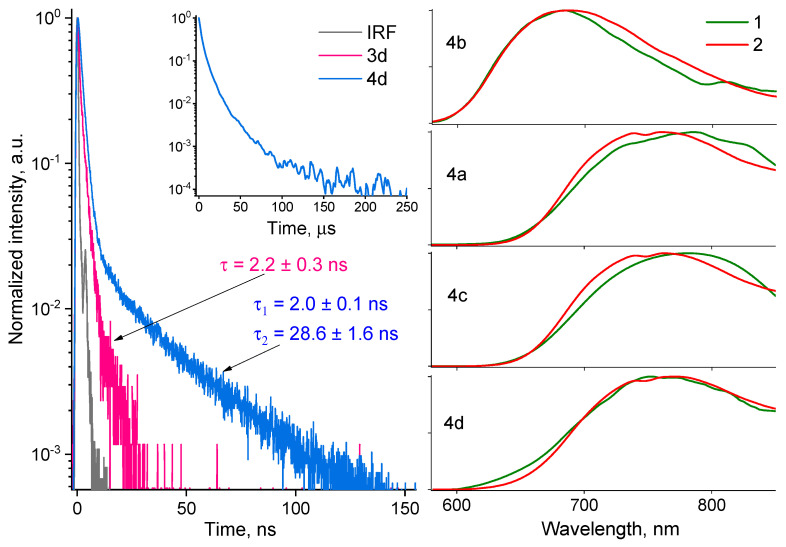
(**Left**): PL decays for the **3d** and **4d** dyes recorded at 700 nm upon laser excitation at 450 nm at 300 K; (**right**): room temperature phosphorescence (1) recorded with a time delay of 5 us and steady-state luminescence (2) spectra for furazan-based dyes upon excitation at 450 nm. All the dyes were dissolved in toluene with a concentration of about 10^−5^ M.

Since PL decays for the **3d** and **4d** molecules demonstrate mono- and bi-exponential behaviors, respectively, with lifetimes relatively close to the lifetimes (τ) of the fast-time component, we have assumed that the dyes have different emission characteristics (see Figure 8, left). In the inset, one can see the biexponential PL decay of the toluene-dissolved **4d** dye recorded within the time window of 250 μs, which demonstrates a weighted average lifetime of about 11 μs. In particular, the furoxan-based dye demonstrates pure fluorescence and later demonstrates both fluorescence and room temperature phosphorescence (RTP). To prove our assumption, we have measured time-resolved luminescence spectra with a delay of 5 μs at 300 K for all investigated dyes (see Figure 8, right). As a result, we observe RTP at the same spectral range as luminescence for the **4d**, **4a**, **4c**, and **4d** dyes (see Figure 8). Similar PL decays and relatively close lifetimes are observed for the **4a**, **4c** and **4d** dyes. In addition, at 77 K, one can see the well-pronounced rise-time component for the **4d** dye within the initial 30 ns in the PL decay associated with the population of the triplet state (see Figure S12). For furoxan-based dyes, we observe no RTP. Thus, the emission maxima of **4d**, **4a**, **4c**, and **4d** are red-shifted in comparison to furoxan analogs due to intensive RTP.

The PL quantum yields (Φ) estimated for all the investigated dyes dissolved in toluene and THF using the absolute method are provided in Table 2. The relatively low polarity of toluene mitigates PL quenching and provides higher Φ values. The highest Φ values are achieved for **4a**, **4c**, and **4d** (2.2%, 2.8%, and 2.0%, respectively). Since the UV-Vis spectra for the furazan- and furoxan-based compounds with the same donor fragments are quite similar, we conclude that the additional oxygen does not significantly affect the ICT process in the dyes. In our opinion, the strong decrease in luminescence efficiency is probably related to the formation of OH–hydrogen bonds between the dyes and solvents. However, the ICT absorption maxima slightly differ by ~10 nm due to ground state geometry rearrangement caused by steric repulsion between the donor fragment and furoxan’s oxygen. To summarize, both the hydrogen bond formation and repulsion of the furoxan oxygen atom from the electron density of the carbazole can result in fluorescence suppression.

To explore the impact of **3d**·½CHCl_3_ on photophysical properties, we measured the PL spectra of the crystalline powders of the **3d** and **4d** dyes (see Figures S11 and S13). In contrast to the PL of the corresponding solutions, the red-shift in emission maximum is not observed (see Table 2). Moreover, Φ cannot be obtained for **3d** even in the crystalline powder state. Since Φ for the **4d** crystal is 1.1% which is four times higher than that for toluene-dissolved **4d**, we consider that the presence of ½CHCl_3_ plays a crucial role in energy transfer, and as a result, it leads to emission quenching and a decrease in luminescence efficiency.

Within the framework of the research, we conducted a comprehensive study of the influence of the chalcogen in the acceptor fragment on the photophysical properties of D-A molecules (Table 3). We compared the results obtained for oxygen-containing molecules with sulfur and selenium-containing analogs investigated in our previous works [11,14]. The photophysical properties of furazan dyes are insignificantly different from molecules containing 1,2,5-thiadiazolo[3,4-*d*]pyridazine as an acceptor with the same donor fragments [14]. The emission maxima of the investigated dyes blue-shift by 15 nm in comparison to the ones for sulfur molecules. On the contrary, the luminescence spectra of molecules containing selenium in the acceptor differ greatly from the PL spectra of dyes under study. Firstly, the use of furoxan and furazan fragments as acceptors leads to the complete suppression of the phosphorescence band at room temperature in the near-infrared (NIR) spectral region [11]. Simultaneously, the luminescence band appears in the visible spectra region for molecules **4a** and **4c** (λ_em_ = 761–778 nm), which is not observed for similar selenium dyes with the same donors. In addition, the absorption spectra of the studied compounds are blue-shifted by 40–80 nm compared to selenium-containing analogs.

To summarize, the chalcogen variation in the acceptor moiety plays a crucial role in energy migration pathways. In particular, the substitution of selenium by oxygen in the acceptor moiety switches the relaxation process from dominant triplet phosphorescence to pure fluorescence. Moreover, the absorption and emission maxima positions are tuned by the variation of only one atom (O, S, and Se) in the acceptor of D-A-D molecules.

## 3. Materials and Methods

### 3.1. Materials and Reagents

Chemicals were purchased from commercial sources and used as received. 4,7-Dichloro[1,2,5]oxadiazolo[3,4-*d*]pyridazine 1-oxide **1** [15], 1,2,3,3a,4,8b-hexahydrocyclopenta[*b*]indole **2a** [16], 2,3,4,4a,9,9a-hexahydro-1*H*-carbazole **2b** [17], and 2,3,4,4a,9,9a-hexahydro-1*H*-1,4-methanocarbazole **2c** [18] were prepared according to the previously described protocols. All synthetic operations were performed under a dry argon atmosphere. Solvents were purified via distillation over the appropriate drying agents.

### 3.2. Analytical Instruments

^1^H and ^13^C NMR spectra were taken with a Bruker AM-300 machine (Bruker AXS Handheld Inc., Kennewick, WA, USA) at frequencies of 300.1 and 75.5 MHz or a Bruker AV600 machine (Bruker AXS Handheld Inc., Kennewick, WA, USA) at frequencies of 600.1 and 150.9 MHz with TMS as the standard. MS spectra (EI, 70 eV) were obtained with a Finnigan MAT INCOS 50 instrument (Hazlet, NJ, USA). High-resolution MS spectra were measured on a Bruker MICROTOF II instrument (Bruker Daltonik Gmbh, Bremen, Germany) using electrospray ionization (ESI). The measurement was operated in a positive ion mode (interface capillary voltage –4500 V) or in a negative ion mode (3200 V); the mass range was from *m*/*z* 50 to 3000 Da; external or internal calibration was performed with the Electrospray Calibrant Solution (Fluka, Buchs, Switzerland). A syringe injection was used for solutions in acetonitrile, methanol, or water (a flow rate of 3 μL/min). Nitrogen was applied as a dry gas; the interface temperature was set at 180 °C. IR spectra were measured with a Bruker “Alpha-T” instrument in KBr pellets. The UV–Vis spectra of the investigated compounds dissolved in tetrahydrofuran (HPLC SuperGradient, Panreac, Spain) were recorded with a JASCO V-770 spectrophotometer (JASCO, Easton, MD, USA) operating within 200–2500 nm. The concentration of the solutions was about 10^−5^ M. The measurements were performed using quartz cells with a 1 cm path length. Excitation spectra and photoluminescence spectra were obtained using a Horiba Fluorolog QM spectrofluorometer (GMP, Fallanden, Switzerland) with a 75 W xenon arc lamp as the excitation source and a R13456 (Hamamatsu, Hamamatsu City, Japan) photomultiplier tube sensitive in the 200–980 nm emission range as the detector. The photoluminescence quantum yields were measured for solid samples via an absolute method using the same experimental setup equipped with a G8 integration sphere. The PL decays recorded within the time window on the order of hundreds of nanoseconds were obtained via TCSPC method, while the longer ones were measured using the single-shot transient digitizer (SSTD). The laser excitation wavelength is 500 nm.

X-ray diffraction data for **3d** and **4d** were collected at 100K on a four-circle Rigaku XtaLAB Synergy-S diffractometer equipped with an HyPix6000HE area detector (kappa geometry, shutterless ω-scan technique) using graphite-monochromatized Cu Kα radiation (λ = 1.54184 Å). The intensity data were integrated and corrected for absorption and decay using the CrysAlisPro program (version 1.171.42.89a, 2023) [19]. The structures were solved via dual methods using SHELXT-2014/5 [20] and refined via the full-matrix least-squares minimization method on *F*^2^ using SHELXL-2018/3 [21] in the OLEX2 program [22]. All non-hydrogen atoms were refined with individual anisotropic displacement parameters. All hydrogen atoms were placed in ideal calculated positions and refined as riding atoms with relative isotropic displacement parameters. The crystal data, data collection, and structure refinement details are summarized in Table S1 of ESI.

### 3.3. Experimental Details

#### 3.3.1. General Procedure for the Preparation of Bis-Aminated Products **3**(**a**–**c**)

Amine **2**(**a**–**c**) (0.68 mmol) was added to a solution of 4,7-dichloro-[1,2,5]oxadiazolo[3,4-*d*]pyridazine 1-oxide **1** (35 mg, 0.17 mmol) in dry DMF (10 mL) at room temperature with stirring. The mixture was stirred at room temperature for 16 h. The mixture was poured into water (10 mL) and extracted with CH_2_Cl_2_ (3 × 35mL). The combined organic layers were washed with brine, dried over MgSO_4_, filtered, and concentrated under reduced pressure. The crude product was purified using column chromatography (Silica gel Merck 60).

4,7-Bis(2,3,3a,8b-tetrahydrocyclopenta[*b*]indol-4(1*H*)-yl)-[1,2,5]oxadiazolo[3,4-*d*]pyridazine 1-oxide **3a**

Violet solid, 51 mg (67%), and R_f_ = 0.4 (CH_2_Cl_2_). Mp = 196–198 °C. Eluent–CH_2_Cl_2_/hexane at a 1:1 (*v*/*v*) ratio. IR *ν*_max_ (KBr, cm^−1^): 2952, 2863, 1613, 1508, 1484, 1451, 1402, 1264, 1219, 1158, 1099, 1025, 892, 749, 618, 569. ^1^H NMR (300 MHz, CDCl_3_): *δ* 8.63 (dd, *J* = 8.1, 4.8 Hz, 1H), 7.29–7.17 (m, 3H), 7.15–7.05 (m, 2H), 7.04–6.95 (m, 1H), 6.92 (t, *J* = 7.3 Hz, 1H), 5.73–5.63 (m, 1H), 5.47–5.37 (m, 1H), 4.09 (t, *J* = 7.5 Hz, 1H), 3.94–3.84 (m, 1H), 2.22–1.92 (m, 4H), 1.91–1.86 (m, 2H), 1.83–1.40 (m, 6H). ^13^C NMR (75 MHz, CDCl_3_): *δ* 145.1, 143.8, 143.6, 143.2, 137.4, 136.0, 135.3, 127.8, 127.3, 124.7, 124.7, 124.1, 122.1, 117.8, 111.8, 106.4, 68.8, 67.2, 46.2, 46.1, 36.7, 34.5, 34.1, 33.8, 24.4, 23.9. HRMS (ESI-TOF), *m*/*z*: calcd for C_26_H_25_N_6_O_2_ [M + H]^+^, 453.2034, found, 453.2031.

4,7-Bis(1,2,3,4,4a,9a-hexahydro-9*H*-carbazol-9-yl)-[1,2,5]oxadiazolo[3,4-*d*]pyridazine 1-oxide **3b**

Violet solid, 53 mg (66%), and R_f_ = 0.4 (CH_2_Cl_2_). Mp = 188–190 °C. Eluent–CH_2_Cl_2_/hexane at a 1:1 (*v*/*v*) ratio. IR *ν*_max_ (KBr, cm^−1^): 2928, 2856, 1619, 1509, 1483, 1454, 1273, 1121, 1159, 1107, 1026, 752, 650. ^1^H NMR (300 MHz, CDCl_3_): *δ* 8.57 (dd, *J* = 7.9, 3.2 Hz, 1H), 7.31–7.25 (m, 2H), 7.21 (d, *J* = 7.3 Hz, 1H), 7.16–7.07 (m, 2H), 6.95 (t, *J* = 7.1 Hz, 1H), 6.89 (d, *J* = 7.9 Hz, 1H), 5.46–5.34 (m, 1H), 4.69–4.61 (m, 1H), 3.71–3.64 (m, 1H), 3.41–3.33 (m, 1H), 2.48–2.40 (m, 1H), 2.25–2.11 (m, 1H), 2.01–1.83 (m, 4H), 1.70–1.60 (m, 2H), 1.48–1.28 (m, 8H). ^13^C NMR (75 MHz, CDCl_3_): *δ* 145.9, 144.5, 143.5, 143.4, 138.4, 135.6, 134.5, 128.2, 127.7, 124.7, 124.0, 123.2, 122.6, 119.9, 112.7, 106.9, 65.3, 64.0, 41.3, 41.1, 30.4, 29.0, 28.0, 26.9, 23.3, 22.9, 22.3, 21.6. HRMS (ESI-TOF), *m*/*z*: calcd for C_28_H_29_N_6_O_2_ [M + H]^+^, 481.2347, found, 481.2332.

4,7-Bis(1,2,3,4,4a,9a-hexahydro-9*H*-1,4-methanocarbazol-9-yl)-[1,2,5]oxadiazolo[3,4-*d*]pyridazine 1-oxide **3c**

Violet solid, 57 mg (67%), and R_f_ = 0.4 (CH_2_Cl_2_). Mp = 196–198 °C. Eluent–CH_2_Cl_2_/hexane at a 1:1 (*v*/*v*) ratio. IR ν_max_ (KBr, cm^−1^): 2954, 2873, 1610, 1507, 1482, 1447, 1405, 1349, 1254, 1176, 1112, 1026, 908, 750, 730, 619. ^1^H NMR (300 MHz, CDCl_3_): *δ* 8.64 (dd, *J* = 12.7, 8.2 Hz, 1H), 7.25–7.00 (m, 6H), 6.88 (t, *J* = 7.0 Hz, 1H), 5.13 (dd, *J* = 15.4, 7.9 Hz, 1H), 4.99–4.93 (m, 1H), 3.54 (d, *J* = 7.9 Hz, 1H), 3.34 (d, *J* = 8.1 Hz, 1H), 2.59–2.42 (m, 2H), 2.40–2.15 (m, 2H), 1.74–1.30 (m, 10H), 1.17–1.08 (m, 2H). ^13^C NMR (75 MHz, CDCl_3_): δ 146.5, 145.1, 144.1, 143.2, 138.0, 134.9, 133.8, 127.8, 127.4, 124.8, 124.2, 123.7, 121.8, 117.1, 112.3, 105.8, 69.8, 69.3, 51.0, 50.9, 44.0, 43.4, 43.1, 42.0, 32.3, 32.0, 28.6, 28.0, 25.5, 25.1. HRMS (ESI-TOF), *m*/*z*: calcd for C_30_H_29_N_6_O_2_ [M + H]^+^, 505.2347, found, 505.2344.

For 4,7-Di(9*H*-carbazol-9-yl)-[1,2,5]oxadiazolo[3,4-*d*]pyridazine 1-oxide **3d**, 60% sodium powered in mineral oil (12 mg, 0.48 mmol) was added to a solution of carbazole **2d** (80 mg, 0.48 mmol) in dry DMF (10 mL) at room temperature with stirring. The reaction mixture was stirred for 30 min; then, 4,7-dichloro-[1,2,5]oxadiazolo[3,4-*d*]pyridazine 1-oxide **1** (50 mg, 0.24 mmol) was added. The mixture was stirred for 12 h at room temperature. The reaction mixture was treated in the same way as in the general procedure for the preparation of compounds **3a**–**c**. The crude product was purified via column chromatography (Silica gel Merck 60, eluent–CH_2_Cl_2_/Hexane at a 1:1 (*v*/*v*) ratio) to afford 35 mg (70%) of target compound **3d** as a dark red solid, with R_f_ = 0.3 (CH_2_Cl_2_/Hexane = 2:1(*v*/*v*)). Mp. = 280–283 °C. IR *ν*_max_ (KBr, cm^−1^): 1629, 1609, 1492, 1458, 1334, 1297, 1243, 1218, 1150, 1008, 746, 719, 591. ^1^H NMR (300 MHz, CDCl_3_, ppm): 8.15–8.12 (m, 4H), 8.05 (d, *J* = 8.2 Hz, 2H), 7.66 (d, *J* = 8.2 Hz, 2H), 7.54–7.40 (m, 8H). ^13^C NMR (75 MHz, CDCl_3_, ppm): δ 144.9, 143.0, 139.2, 138.89, 138.86, 126.9, 126.7, 125.9, 125.3, 123.3, 123.2, 120.6, 120.3, 113.4, 111.6, 106.7. HRMS (ESI-TOF), *m*/*z*: found, 469.1395; calcd for C_28_H_17_N_6_O_2_ [M + H]^+^, 469.1408.

#### 3.3.2. General Procedure for the Preparation of Di-Aminated Products **4**

Bis-aminated products **3a**–**c** (0.2 mmol) were added with stirring to a solution of Ph_3_P (62 mg, 0.24 mmol) in dry CH_2_Cl_2_ (10 mL). The mixture was stirred at room temperature for 3 h. Then, the mixture was poured into water (25 mL) and extracted with EtOAc (3 × 35mL). The combined organic layers were washed with brine, dried over MgSO_4_, filtered, and concentrated under reduced pressure. The crude product was purified using column chromatography (Silica gel Merck 60).

4,7-Bis(2,3,3a,8b-tetrahydrocyclopenta[*b*]indol-4(1*H*)-yl)-[1,2,5]oxadiazolo[3,4-*d*]pyridazine **4a**

Violet solid, 40 mg (72%), and R_f_ = 0.4 (CH_2_Cl_2_). Mp = 178–180 °C. Eluent–CH_2_Cl_2_/hexane at a 1:1 (*v*/*v*) ratio. IR *ν*_max_ (KBr, cm^−1^): 2955, 2863, 1487, 1453, 1421, 1349, 1215, 894, 749, 666, 572. ^1^H NMR (300 MHz, CDCl_3_): 8.62 (d, *J* = 7.8 Hz, 2H), 7.29–7.19 (m, 4H), 7.02 (t, *J* = 6.9 Hz, 2H), 5.70–5.61 (m, 2H), 4.13–4.04 (m, 2H), 2.22–2.01 (m, 6H), 1.84–1.63 (m, 4H), 1.55–1.40 (m, 2H). ^13^C NMR (75 MHz, CDCl_3_): *δ* 144.2, 141.4, 141.3, 135.6, 127.7, 124.0, 123.1, 116.4, 67.4, 45.9, 36.3, 34.5, 23.9. HRMS (ESI-TOF), *m*/*z*: calcd for C_26_H_25_N_6_O [M + H]^+^, 437.2084, found, 437.2068.

4,7-Bis(1,2,3,4,4a,9a-hexahydro-9*H*-carbazol-9-yl)-[1,2,5]oxadiazolo[3,4-*d*]pyridazine **4b**

A yield of 144 mg (65%), violet solid, and R_f_ = 0.3 (a hexane–CH_2_Cl_2_ ratio of 2:1, *v*/*v*). Mp = 144–146 °C. IR *ν*_max_ (KBr, cm^−1^): 2929, 2855, 1482, 1454, 1424, 1271, 1214, 1164, 1094, 1020, 971, 927, 886, 847, 752, 689, 576. ^1^H NMR (300 MHz, CDCl_3_): δ 8.55 (d, *J* = 8.0, 2H), 7.33 (t, *J* = 8.0, 2H), 7.27 (d, *J* = 7.3, 2H), 7.11 (t, *J* = 7.3, 2H), 5.42–5.35 (m, 2H), 3.72–3.66 (m, 2H), 2.43 (d, *J* = 14.5, 2H), 2.13–1.91 (m, 4H), 1.71–1.62 (m, 4H), 1.41–1.31 (m, 6H). ^13^C NMR (75 MHz, CDCl_3_): δ 143.0, 141.2, 141.0, 134.6, 127.5, 123.3, 122.5, 118.2, 63.7, 40.2, 27.7, 24.3, 22.6, 21.0. HRMS (ESI-TOF), *m*/*z*: calcd for C_28_H_29_N_6_O [M + H]^+^, 465.2397, found, 465.2395.

4,7-Bis(1,2,3,4,4a,9a-hexahydro-9*H*-1,4-methanocarbazol-9-yl)-[1,2,5]oxadiazolo[3,4-*d*]pyridazine **4c**

Violet solid, 40 mg (72%), and R_f_ = 0.4 (CH_2_Cl_2_). Mp = 178–180 °C. Eluent–CH_2_Cl_2_/hexane at a 1:1 (*v*/*v*) ratio. IR *ν*_max_ (KBr, cm^−1^): 2953, 2870, 1482, 1451, 1350, 1254, 1173, 891, 748, 693, 648, 566. ^1^H NMR (300 MHz, CDCl_3_): 8.62 (d, *J* = 8.1 Hz, 2H), 7.28–7.17 (m, 4H), 7.00 (t, *J* = 7.3 Hz, 2H), 5.14 (d, *J* = 7.9 Hz, 2H), 3.53 (d, *J* = 7.9 Hz, 2H), 2.41 (d, *J* = 20.1 Hz, 4H), 1.74–1.44 (m, 10H), 1.13 (d, *J* = 10.4 Hz, 2H). ^13^C NMR (75 MHz, CDCl_3_): *δ* 145.4, 141.4, 141.4, 134.6, 127.8, 124.2, 122.9, 116.1, 69.3, 50.7, 43.5, 43.4, 32.0, 28.2, 25.4. HRMS (ESI-TOF), *m*/*z*: calcd for C_30_H_29_N_6_O [M + H]^+^, 489.2397, found, 489.2388.

4,7-Bis(1,2,3,4,4a,9a-hexahydro-9*H*-carbazol-9-yl)-[1,2,5]oxadiazolo[3,4-*d*]pyridazine **4d**

2,3-Dichloro-5,6-dicyano-1,4-benzoquinone (74 mg, 0.32 mmol) was added to a solution of amine **2d** (60 mg, 0.13 mmol) in toluene (12 mL). The mixture was refluxed for 7 h; diluted with EtOAc (30 mL); washed with aq. NaHSO_3_, Na_2_CO_3_, water, and brine; dried over MgSO4; and concentrated under reduced pressure. The crude product was purified using column chromatography (Silica gel Merck 60, eluent–CH_2_Cl_2_/hexane at a 2:1 (*v*/*v*) ratio) to afford 45 mg (78%) of target compound **4d** as a dark red solid, with R_f_ = 0.3 (hexane–CH_2_Cl_2_ = 2:1 *v*/*v*). Mp. = 182–184 °C. IR *ν*_max_ (KBr, cm^−1^): 1493, 1467, 1453, 1334, 1267, 1221, 1122, 744, 718, 675, 609. ^1^H NMR (300 MHz, CDCl_3_): δ 8.16 (d, *J* = 7.3, 4H), 8.07 (d, *J* = 8.1, 4H), 7.53 (td, *J* = 8.1, 1.7, 4H), 7.47 (t, *J* = 7.3, 4H). ^13^C NMR (75 MHz, CDCl_3_): δ 143.1, 142.6, 139.0, 126.8, 125.9, 123.4, 120.3, 113.2. HRMS (ESI-TOF), *m*/*z*: calcd for C_28_H_17_N_6_O [M + H]^+^, 453.1458, found, 453.1445.

### 3.4. DFT Quantum Calculations

DFT calculations were performed using the functional PBE1PBE(PBE0) [23] and the DEF2TZVP [24] basis with the GD3BJ empirical dispersion in the Gaussian 2016 program [25] (gas phase, 298K, see Supplementary Information).

## 4. Conclusions

We have successfully synthesized a series of novel D-A-D dyes based on [1,2,5]oxadiazolo[3,4-*d*]pyridazine and [1,2,5]oxadiazolo[3,4-*d*]pyridazine 1-oxide with various donor fragments. We have established that the emission of furazan-based dyes has the triplet-state phosphorescence nature as well as previously investigated [1,2,5]selenadiazolo[3,4-*d*]pyridazine derivatives. On the contrary, our studies reveal that the furoxan acceptor provides pure fluorescence in the red spectral region. Furazan derivatives demonstrate higher luminescence quantum yields compared to their furoxan analogs. The observed decrease in fluorescence quantum yields for furoxan derivatives can be attributed to two main factors: (1) a decrease in the overall conjugation of the π–electron system due to the steric repulsion between the furoxan’s external oxygen atom and the electron density of the carbazole system and (2) the formation of hydrogen bonds. The results obtained will subsequently help researchers design new high-performance donor–acceptor molecules with tailored luminescence properties. According to the results of X-ray diffraction studies of **3d**·½CHCl_3_ and **4d**, the [1,2,5]oxadiazolo[3,4-*d*]pyridazine heterocycle is not entirely flat, likely due to intrinsic electronic effects rather than intra- and intermolecular van der Waals interactions. Nearly flat *N*-carbazolyl groups are rotated with respect to the N_2_C_4_ plane of the central heterocycle by rather large angles due to intramolecular non-covalent interactions, indicating that π-π conjugation between the C_12_H_8_N and N_4_C_4_O/N_4_C_4_O_2_ heterocycle systems is lost to a substantial extent. Therefore, the *N*-carbazolyl group cannot effectively serve as an antenna to improve the photophysical properties of the dyes. Presumably, the short non-covalent interactions of the furoxan system with a lattice chloroform molecule in **3d** lead to luminescence quenching, whereas intermolecular π-π interactions of the nitrogen atoms of the pyridazine ring with the *N*-carbazolyl group facilitate the intermolecular charge transfer in **4d**.

## Figures and Tables

**Table 1:** Selected bond distances for **3d** and **4d** (Å).

3d (1st Molecule)		3d (2nd Molecule)		4d	
N1-N2	1.383(4)	N7-N8	1.379(4)	N1-N2	1.374(2)
N1-C1	1.308(4)	N8-C32	1.314(4)	N1-C1	1.311(2)
N2-C4	1.311(4)	N7-C29	1.307(4)	N2-C4	1.308(2)
C1-C2 *	1.462(7)	C31-C32 *	1.428(7)	C1-C2	1.450(2)
C2-C3 *	1.417(7)	C30-C31 *	1.402(7)	C2-C3	1.413(3)
C3-C4 *	1.410(8)	C29-C30 *	1.435(6)	C3-C4	1.443(2)
N3-C2 *	1.342(8)	N10-C31 *	1.342(10)	N3-C2	1.320(2)
N4-C3 *	1.309(9)	N9-C30 *	1.327(8)	N4-C3	1.318(2)
O1-N3 *	1.466(6)	O3-N10 *	1.483(5)	O1-N3	1.381(2)
O1-N4 *	1.380(7)	O3-N9 *	1.370(5)	O1-N4	1.373(2)
O2-N3 *	1.213(6)	O4-N10 *	1.209(5)	-	-
N5-C1	1.390(4)	N12-C32	1.387(4)	N5-C1	1.382(2)
N6-C4	1.392(4)	N11-C29	1.388(4)	N6-C4	1.383(2)

* Bond distances for the major parts of the disordered furoxan fragments are listed for **3d**. See ESI for details.

**Table 2:** Calculated bond distances for **3d** and **4d** (Å).

3d		4d	
N1-N2	1.346	N1-N2	1.342
N1-C1	1.304	N1-C1	1.334
N2-C4	1.303	N2-C4	1.334
C1-C2	1.423	C1-C2	1.450
C2-C3	1.410	C2-C3	1.459
C3-C4	1.437	C3-C4	1.451
N3-C2	1.338	N3-C2	1.333
N4-C3	1.309	N4-C3	1.332
O1-N3	1.419	O1-N3	1.372
O1-N4	1.347	O1-N4	1.371
O2-N3	1.198	-	-
N5-C1	1.379	N5-C1	1.417
N6-C4	1.378	N6-C4	1.417

**Table 3:** Photophysical parameters obtained for all dyes: absorption maximum wavelength (λ*_abs_*), maximum molar extinction coefficient (*ε*), oscillator strength (*f*), wavelength of emission maximum (λ*_em_*), full width at half-maximum (FWHM) for the emission spectrum, and Stokes shift (Δ*ν*).

Solvent	λ*_abs_*(LE) nm	λ*_abs_*(ICT) nm	*ε*_max_ × 10^3^ mol × L^−1^ × cm^−1^	*f*	λ*_em_* nm	FWHM nm	Δ*ν* cm^−1^	Φ, %
**3d**								
Toluene	285	532	12.5	0.025	667	137	3800	<0.1
THF	281	510	8.2	0.016	675	200	4800	<0.1
Crystalline powder *	-	-	-	-	745	105	-	<0.1
**4d**								
Toluene	288	531	9.4	0.023	688	156	4300	0.4
THF	280	507	12.0	0.047	706	176	5600	<0.1
Crystalline powder *	-	-	-	-	735	128	-	1.1
**3a**								
Toluene	295	577	10.5	0.018	705	130	4200	<0.1
THF	294	559	21.2	0.012	-	-	-	-
**4a**								
Toluene	290	567	19.4	0.026	761	194	4550	2.2
THF	292	556	7.5	0.015	778	194	5130	1.2
**3c**								
Toluene	291	587	8.3	0.013	705	137	2900	<0.1
THF	291	566	7.4	0.002	772	197	4700	<0.1
**4c**								
Toluene	290	569	8.0	0.013	761	189	4430	2.8
THF	288	556	3.8	0.012	774	186	5100	1.6
**3b**								
Toluene	292	552	8.1	0.022	729	145	4400	<0.1
THF	291	533	7.1	0.019	-	-	-	-
**4b**								
Toluene	291	557	6.7	0.023	767	190	4920	2.0
THF	287	540	22.5	0.035	774	195	5600	0.9

* Luminescence.

## Data Availability

Data are contained within the article and Supplementary Materials.

## Supplementary Materials

The following supporting information can be downloaded at https://www.mdpi.com/article/10.3390/molecules30112374/s1. Characterization data including ^1^H and ^13^C NMR spectra for novel compounds and photoluminescence spectra. The crystal structures of **3d**·½CHCl_3_ and **4d** have been deposited at the Cambridge Crystallographic Data Center with the following reference CCDC numbers: 2401696 (**3d**·½CHCl_3_) and 2401695 (**4d**); they also contain the supplementary crystallographic data. These data can be obtained free of charge from the CCDC via https://www.ccdc.cam.ac.uk/structures/ (accessed on 3 March 2025). References [26,27,28] are cited in the supplementary materials.
