# Supplementary material for: The Effect of the N-Oxide Oxygen Atom on the Crystalline and Photophysical Properties of [1,2,5]Oxadiazolo[3,4-d]pyridazines
Source: Molecules. 2025 May 29;30(11):2374. doi: 10.3390/molecules30112374 (PMC12156411; doi:10.3390/molecules30112374)
Supplement: Supplementary file 1 [file molecules-30-02374-s001.zip › molecules-3641728-supplementary.pdf]

Supplementary Information

Effect of *N*-oxide oxygen atom on the crystalline and photophysical properties of [1,2,5]oxadiazolo[3,4-*d*]pyridazines

Timofey N. Chmovzh[1,2], Alisia V. Tsorieva[3], Vladislav M. Korshunov[3], Egor D. Kotov[1,2,4], Daria I. Nasyrova[1], Mikhail E. Minyaev[1], Nikolay P. Datskevich[3], Ilya V. Taydakov[3], Michail N. Elinson[1]*, and Oleg A. Rakitin[1]*

[1] N. D. Zelinsky Institute of Organic Chemistry, Russian Academy of Sciences, 119991 Moscow, Russia; orakitin@ioc.ac.ru (O.A.R.); elinson@ioc.ac.ru (M.N.E.)

[2] D. I. Mendeleev University of Chemistry and Technology of Russia, Miusskaya sqr., 9, Moscow 125047, Russian Federation; tim1661@yandex.ru (T.N.C.)

[3] P. N. Lebedev Physical Institute of the Russian Academy of Sciences, 119991 Moscow, Russia; tsorievaav@gmail.com (A.V.T.); vladkorshunov@bk.ru (V.M.K.); dac1@yandex.ru (N.P.D); taidakov@gmail.com (I.V.T.)

[4] Skoltech Center for Energy Science and Technology, Skolkovo Institute of Science and Technology, 121205 Moscow, Russian Federation; egor.Kotov.dm@gmail.com (E.D.K.)

* Correspondence: elinson@ioc.ac.ru; Tel.: +7-499-137-3842

Table of Contents

SI-1. ^{1}H and ^{13}C NMR spectra………………………………………………………….. S3

SI-2 Single crystal X-ray diffraction data…………………………………………….. S12

SI-3. Photophysical parameters………………………………………………………… S13

SI-4. Quantum-chemical calculations…………………………………………………… S14

1. ^{1}H and ^{13}C NMR spectra

4,7-Bis(2,3,3a,8b-tetrahydrocyclopenta[b]indol-4(1H)-yl)-[1,2,5]oxadiazolo[3,4-d]pyridazine 1-oxide (3a) ^{1}H NMR(600 MHz)

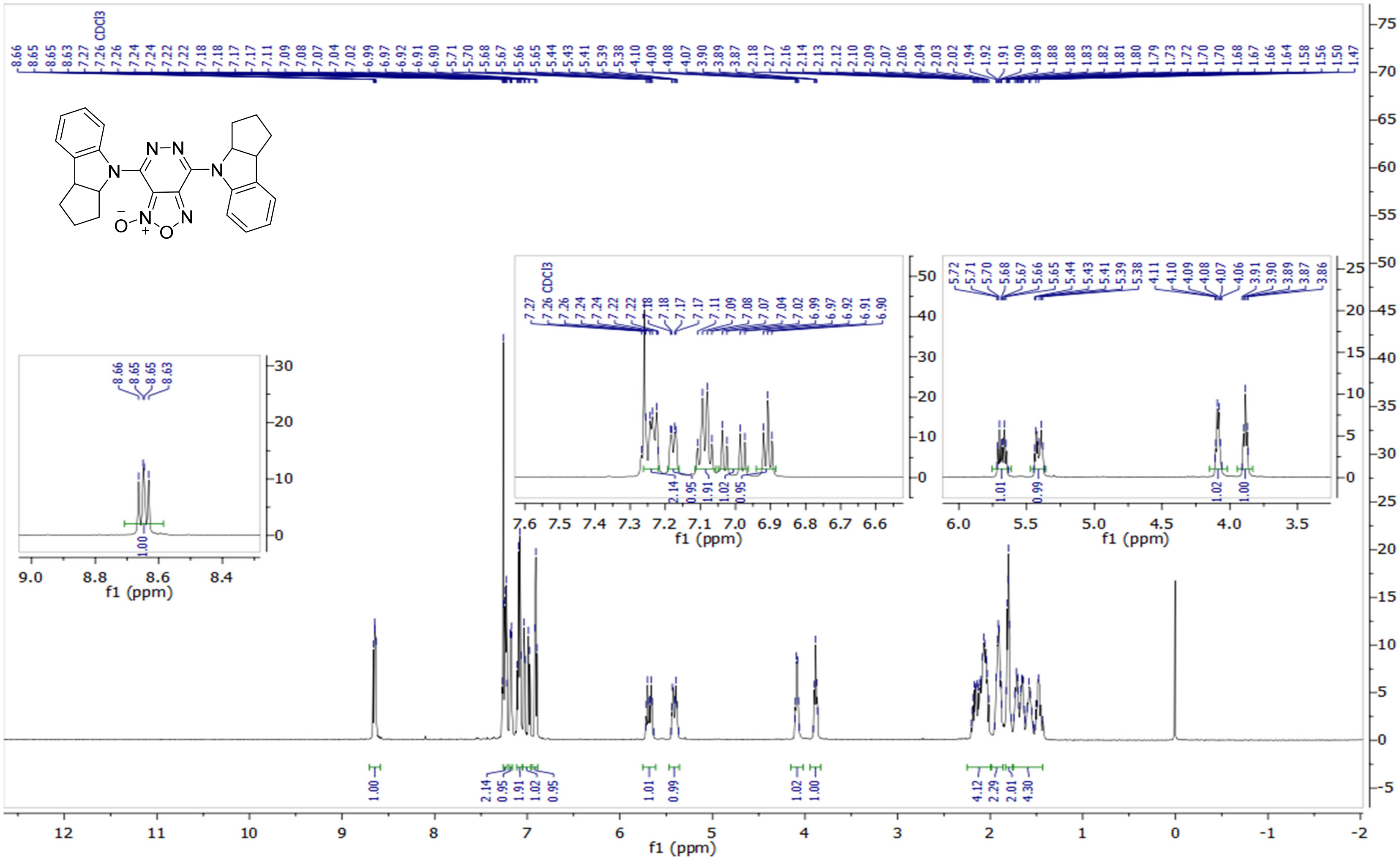

4,7-Bis(2,3,3a,8b-tetrahydrocyclopenta[b]indol-4(1H)-yl)-[1,2,5]oxadiazolo[3,4-d]pyridazine 1-oxide (3a) ^{13}C NMR(150 MHz)

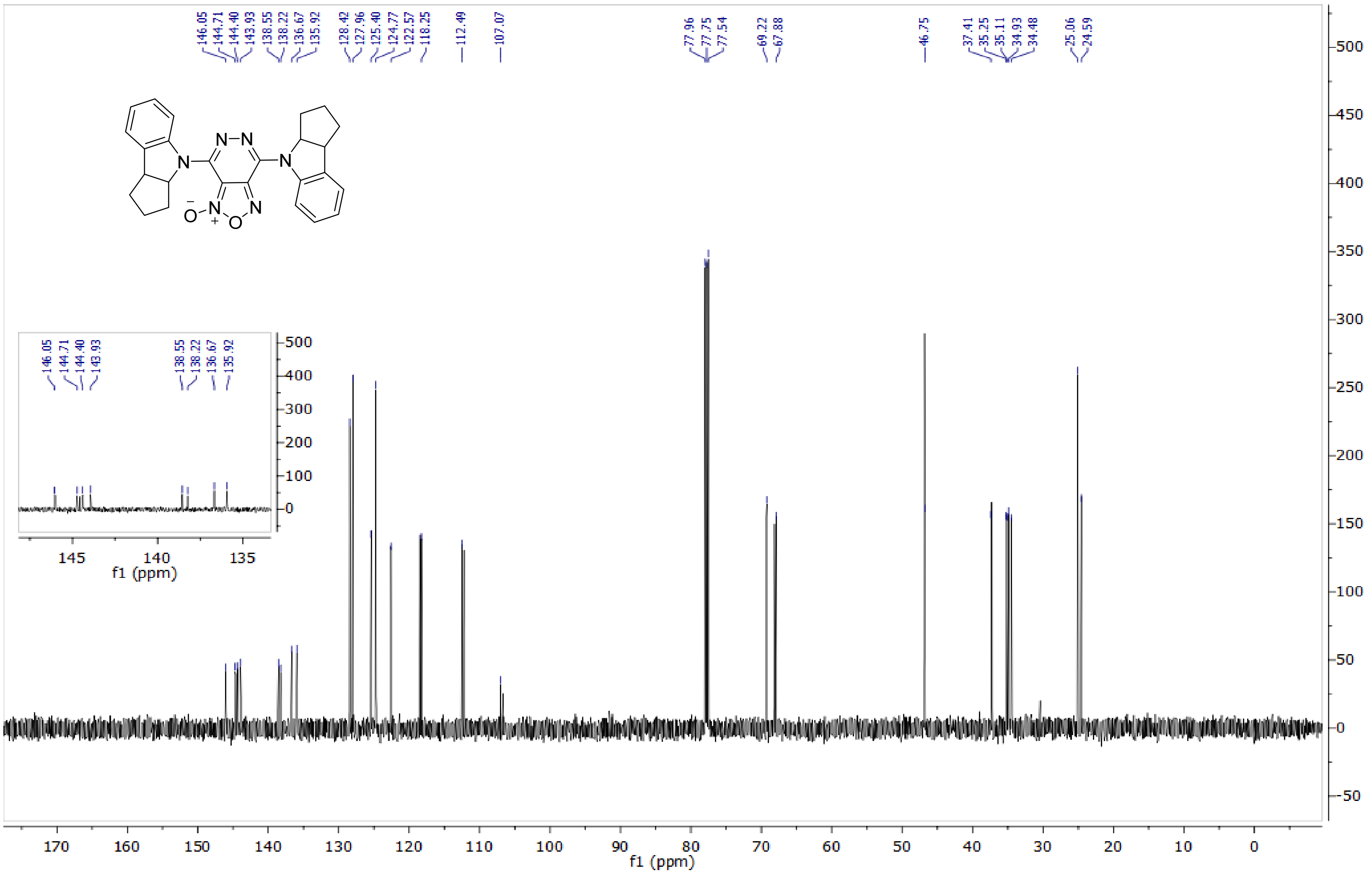

4,7-Bis(1,2,3,4,4a,9a-hexahydro-9H-1,4-methanocarbazol-9-yl)-[1,2,5]oxadiazolo[3,4-d]pyridazine 1-oxide (3c) ^{1}H NMR(600 MHz)

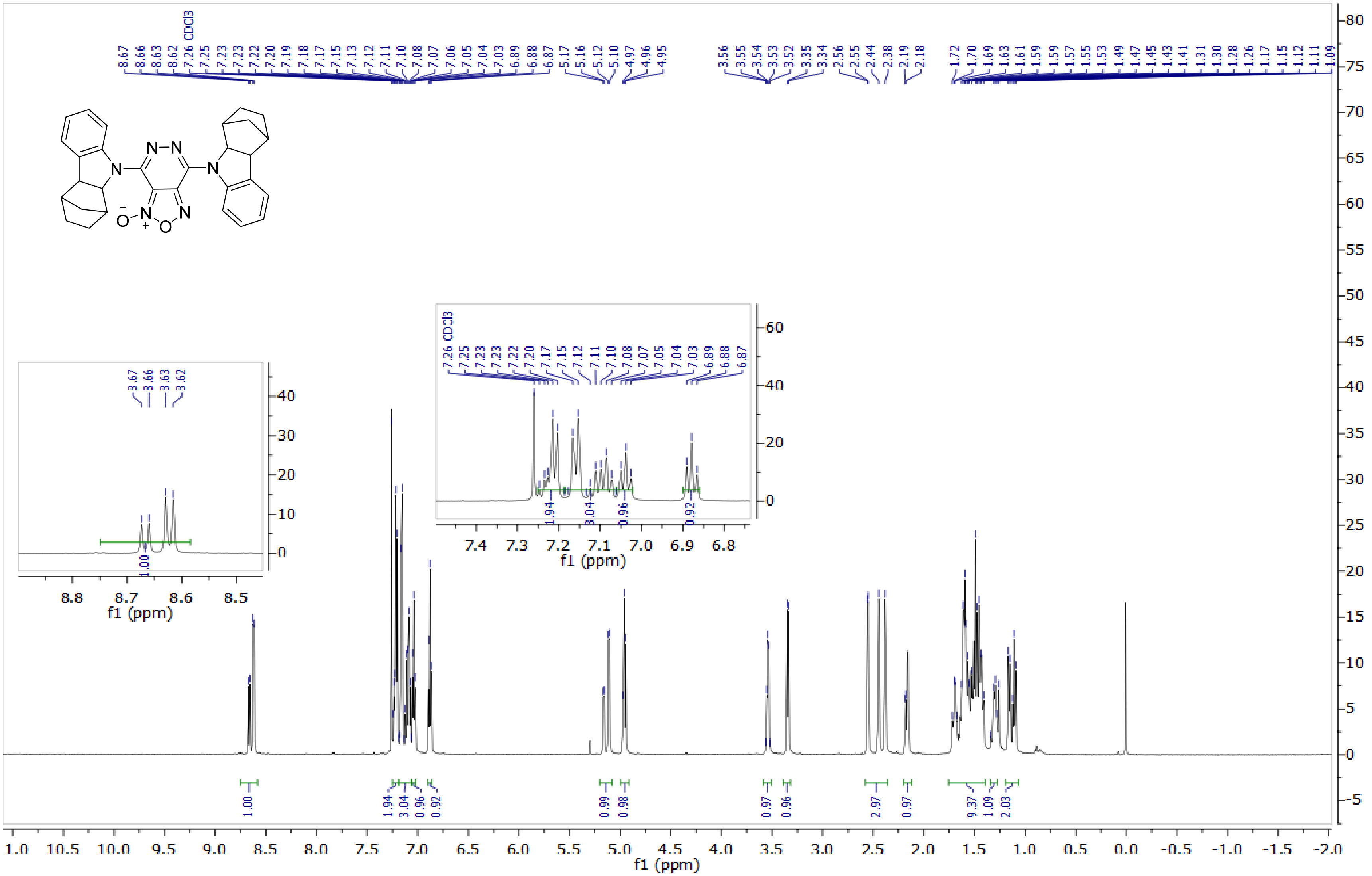

4,7-Bis(1,2,3,4,4a,9a-hexahydro-9H-1,4-methanocarbazol-9-yl)-[1,2,5]oxadiazolo[3,4-d]pyridazine 1-oxide (3c) ^{1}H NMR(150 MHz)

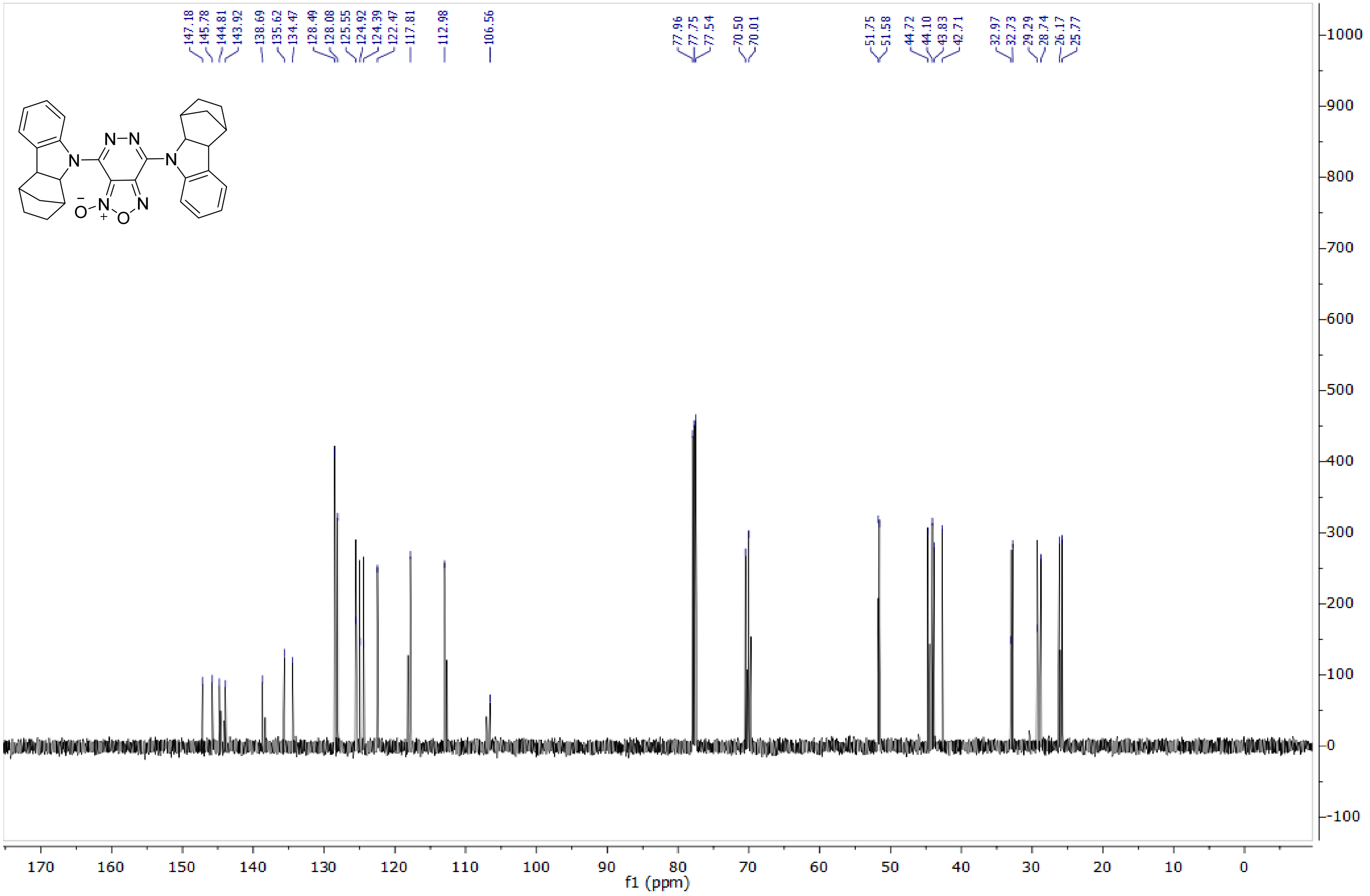

4,7-Di(9H-carbazol-9-yl)-[1,2,5]oxadiazolo[3,4-d]pyridazine 1-oxide (3d) ^{1}H NMR(600 MHz)

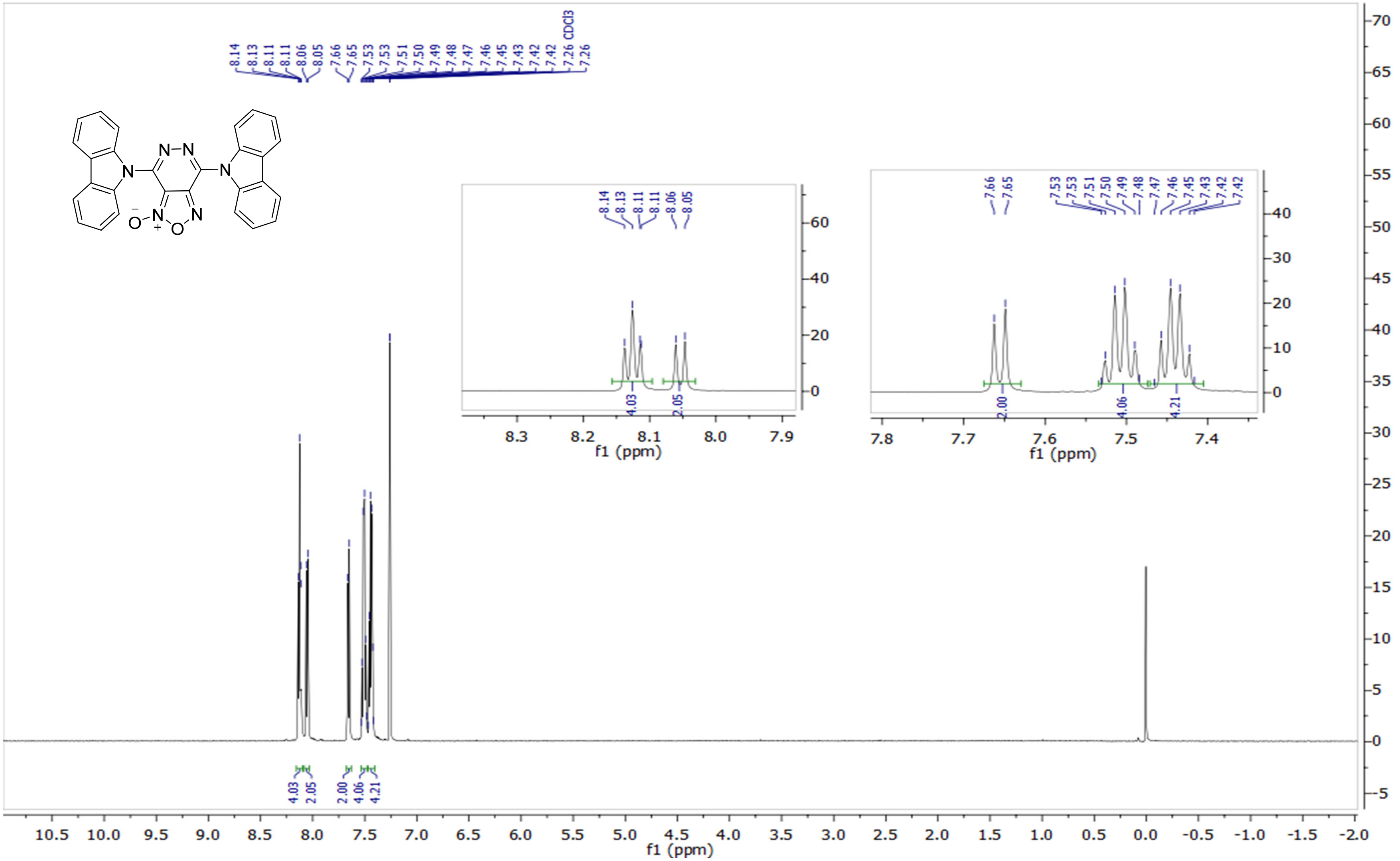

4,7-Di(9H-carbazol-9-yl)-[1,2,5]oxadiazolo[3,4-d]pyridazine 1-oxide (3d) ^{13}C NMR(150 MHz)

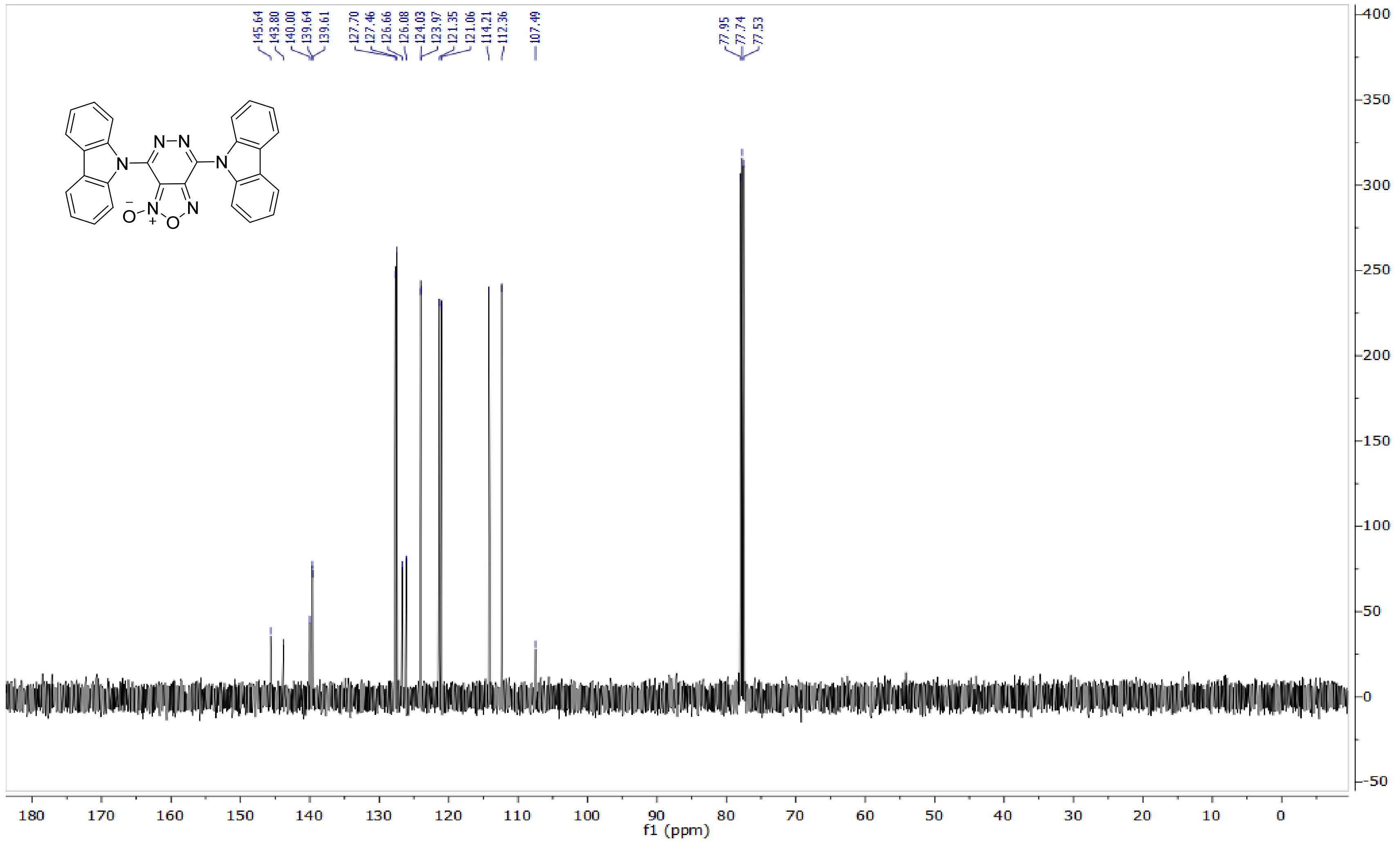

4,7-Bis(2,3,3a,8b-tetrahydrocyclopenta[b]indol-4(1H)-yl)-[1,2,5]oxadiazolo[3,4-d]pyridazine (4a) ^{1}H NMR(300 MHz)

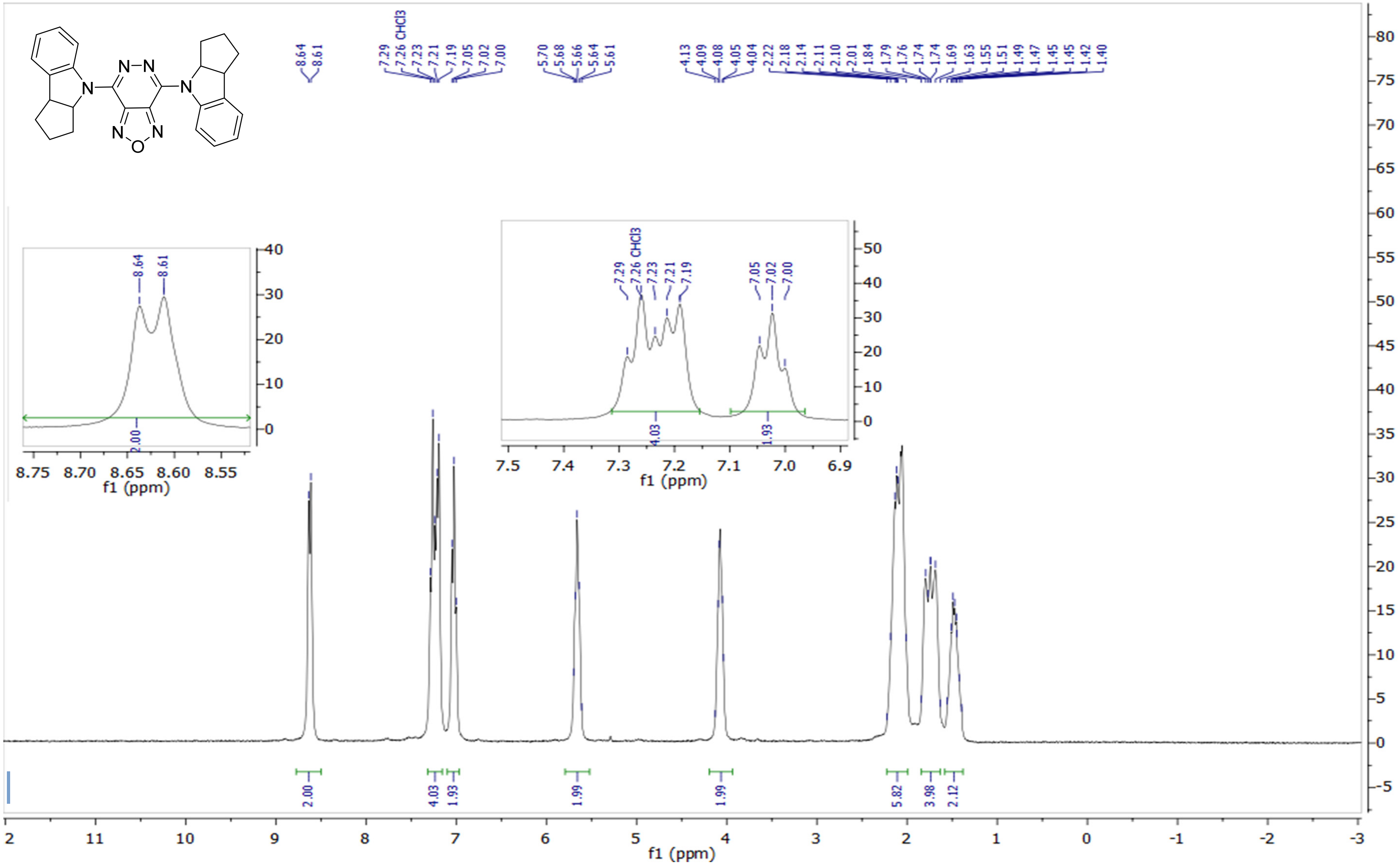

4,7-Bis(2,3,3a,8b-tetrahydrocyclopenta[b]indol-4(1H)-yl)-[1,2,5]oxadiazolo[3,4-d]pyridazine (4a) ^{13}C NMR(75 MHz)

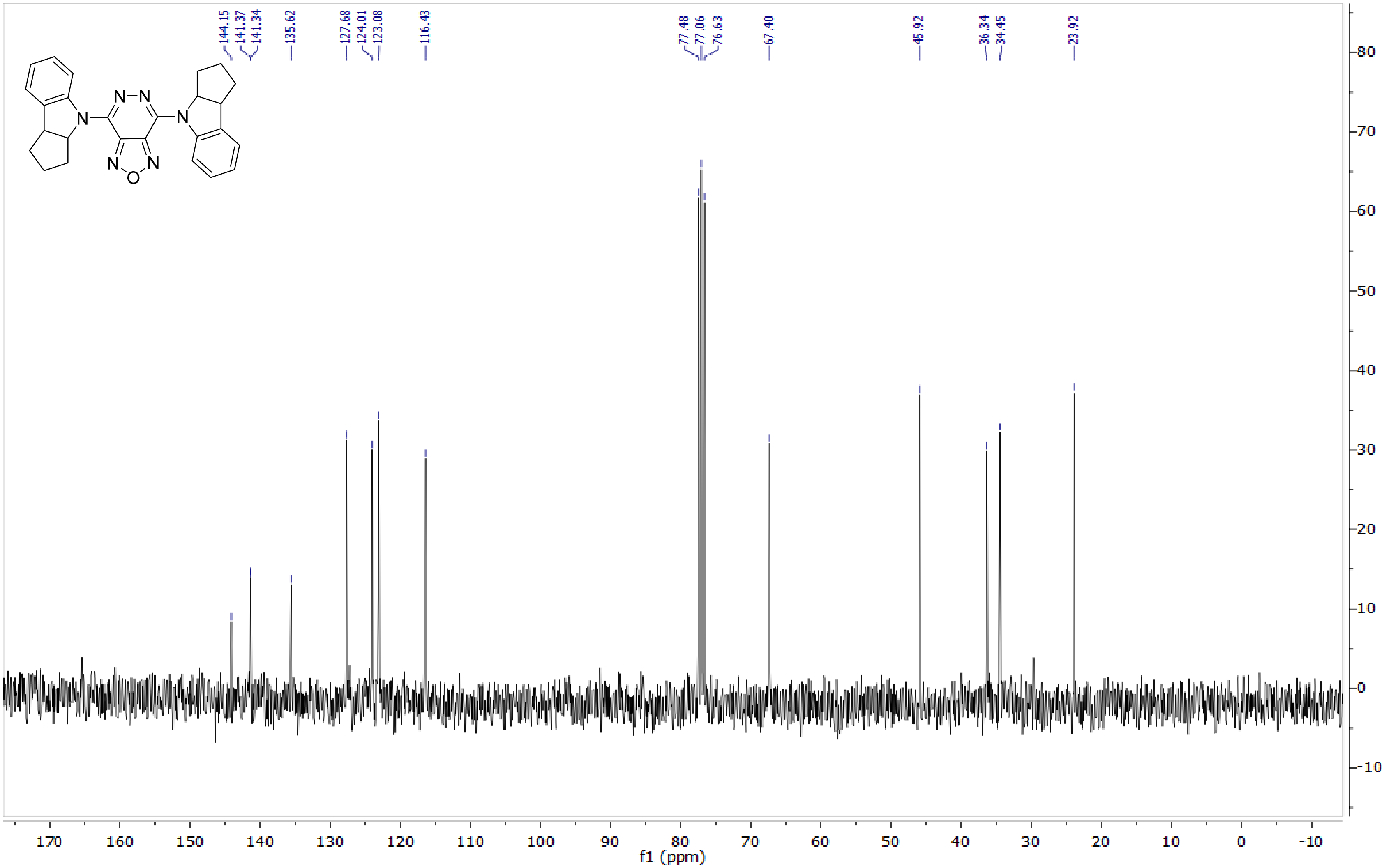

4,7-Bis(1,2,3,4,4a,9a-hexahydro-9H-carbazol-9-yl)-[1,2,5]oxadiazolo[3,4-d]pyridazine (4b) ¹H NMR(300 MHz)

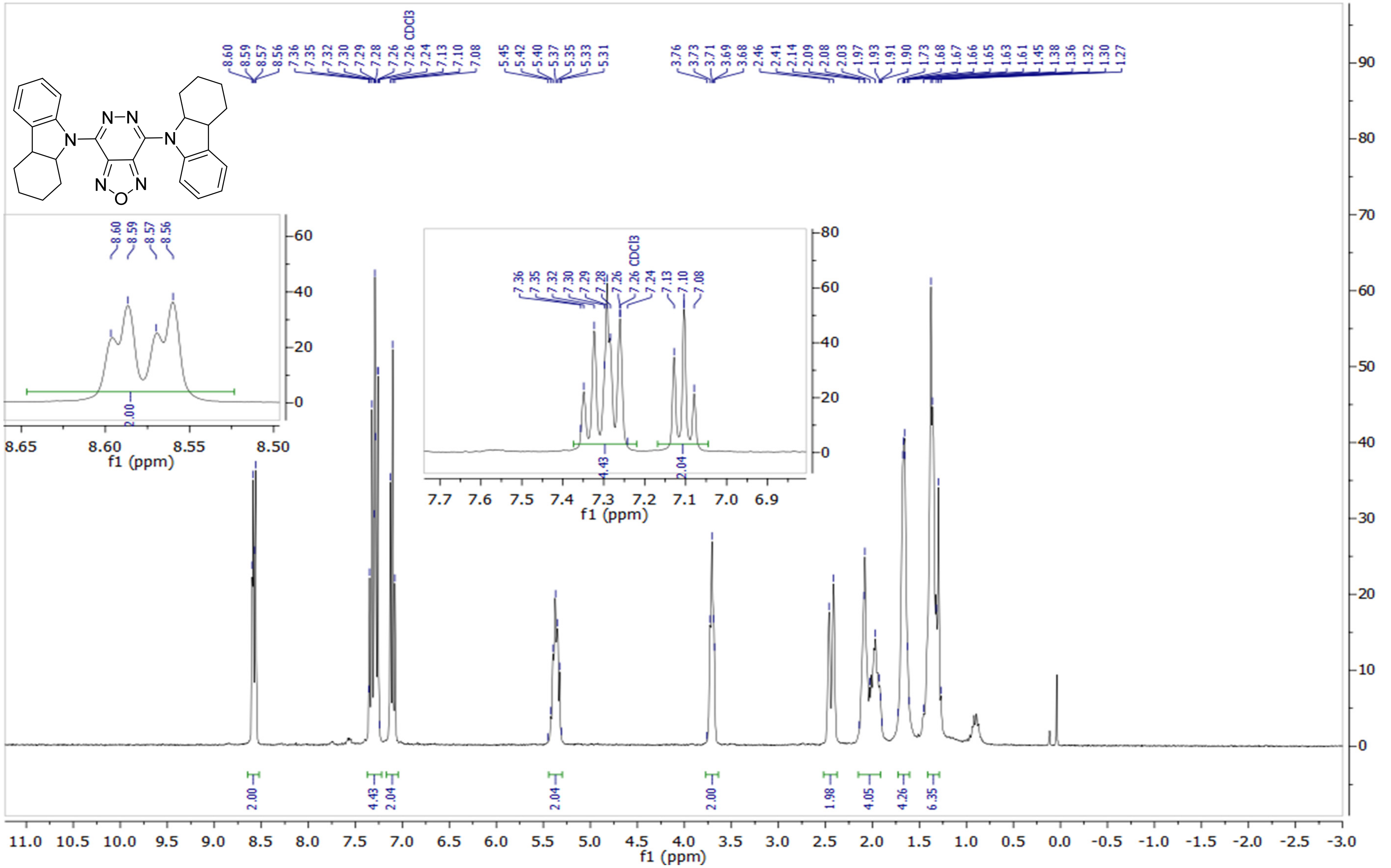

4,7-Bis(1,2,3,4,4a,9a-hexahydro-9H-carbazol-9-yl)-[1,2,5]oxadiazolo[3,4-d]pyridazine (4b) ^{13}C NMR(75 MHz)

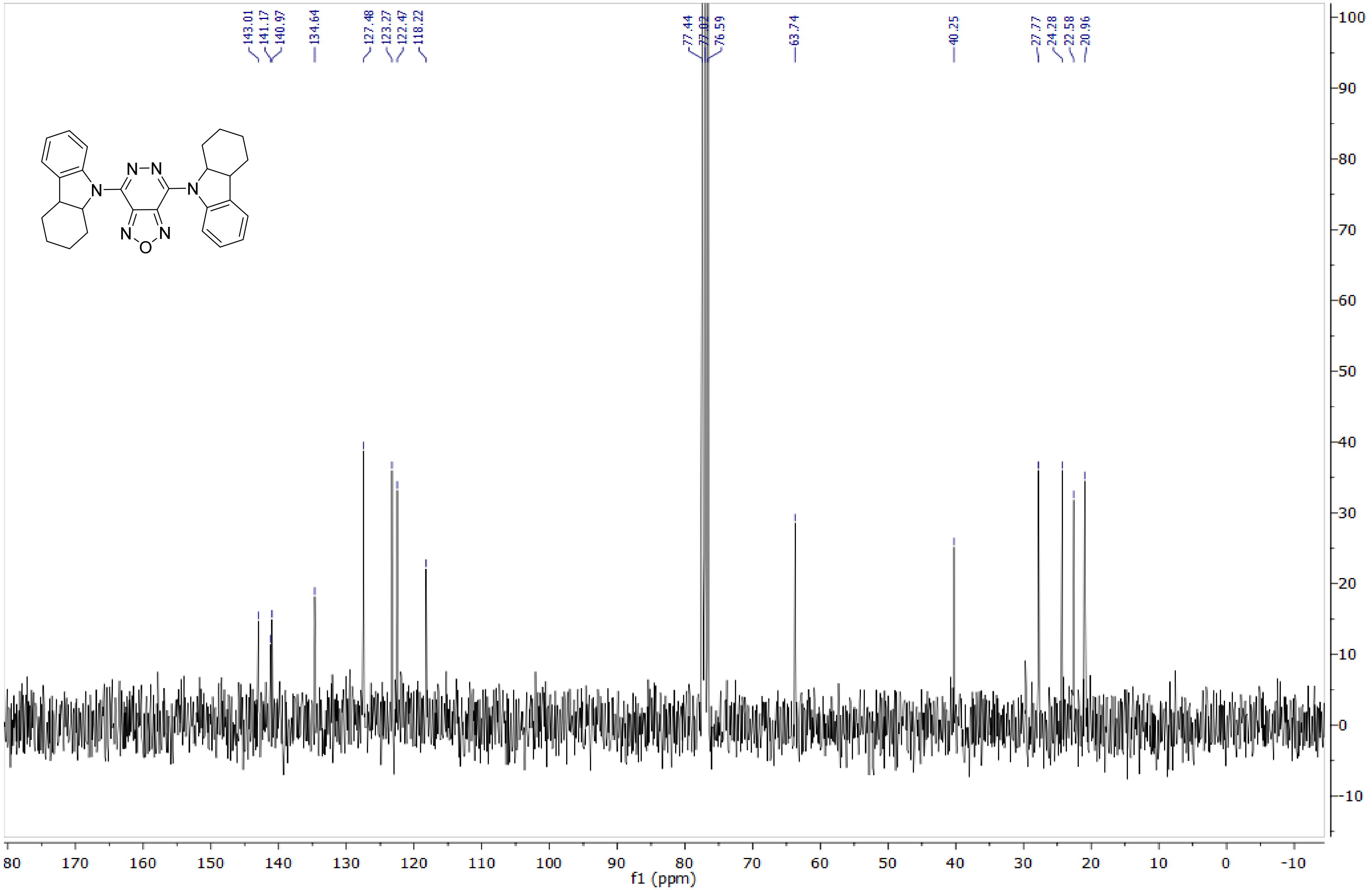

4,7-Bis(1,2,3,4,4a,9a-hexahydro-9H-1,4-methanocarbazol-9-yl)-[1,2,5]oxadiazolo[3,4-d]pyridazine (4c) ^{1}H NMR(300 MHz)

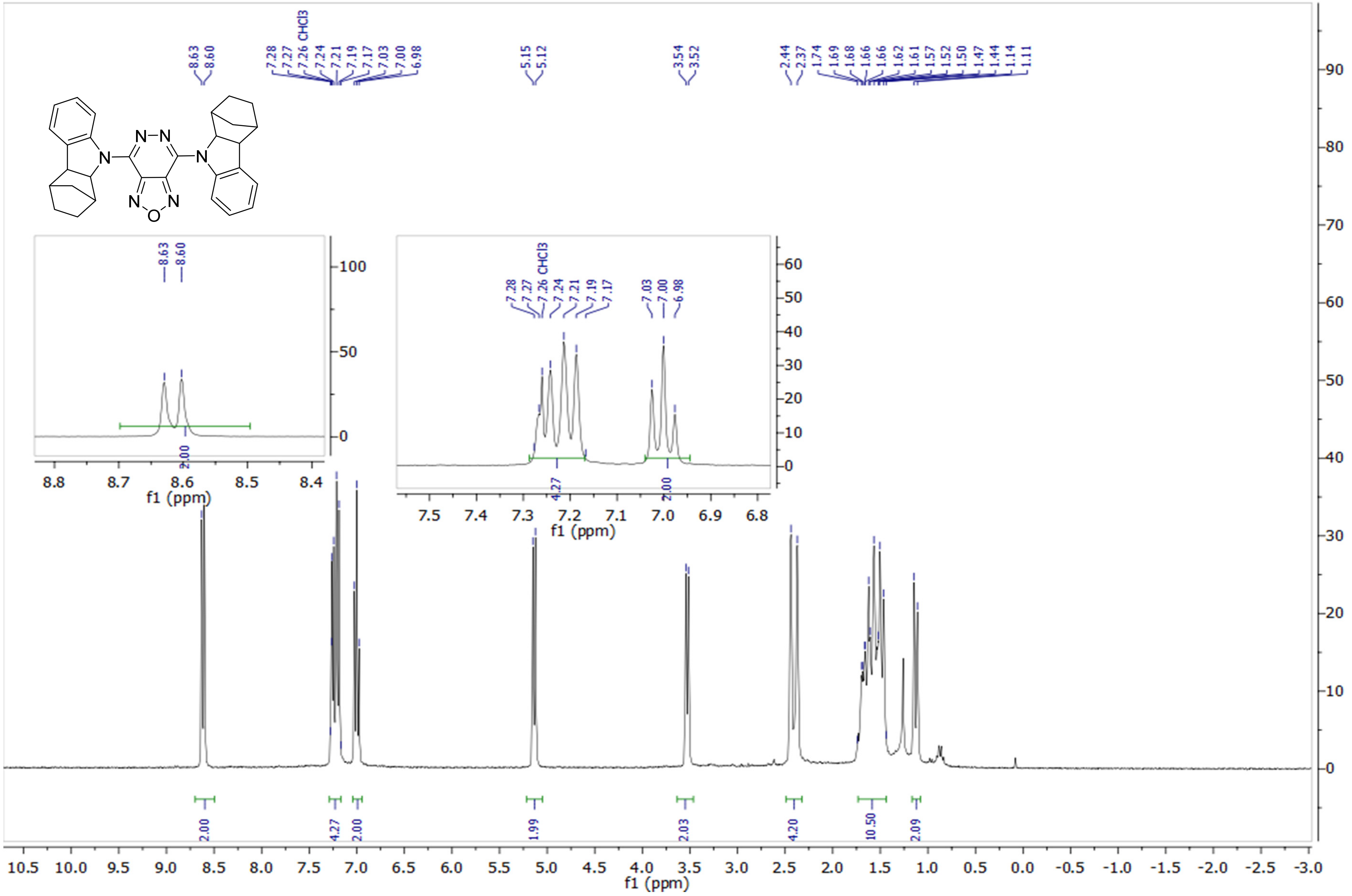

4,7-Bis(1,2,3,4,4a,9a-hexahydro-9H-1,4-methanocarbazol-9-yl)-[1,2,5]oxadiazolo[3,4-d]pyridazine (4c) ^{13}C NMR(75 MHz)

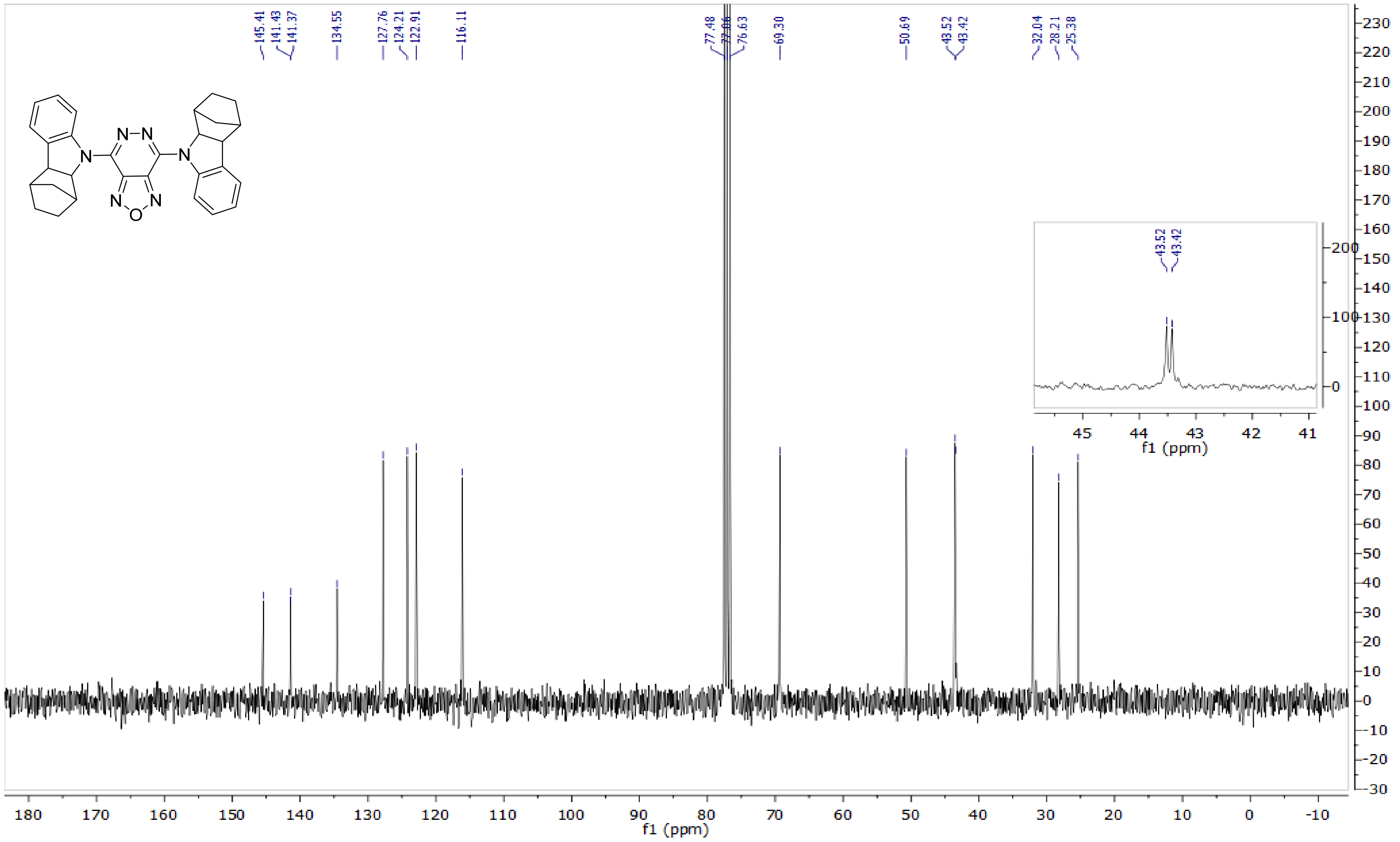

4,7-Bis(1,2,3,4,4a,9a-hexahydro-9H-carbazol-9-yl)-[1,2,5]oxadiazolo[3,4-d]pyridazine (4d) ^{1}**H NMR(300 MHz)**

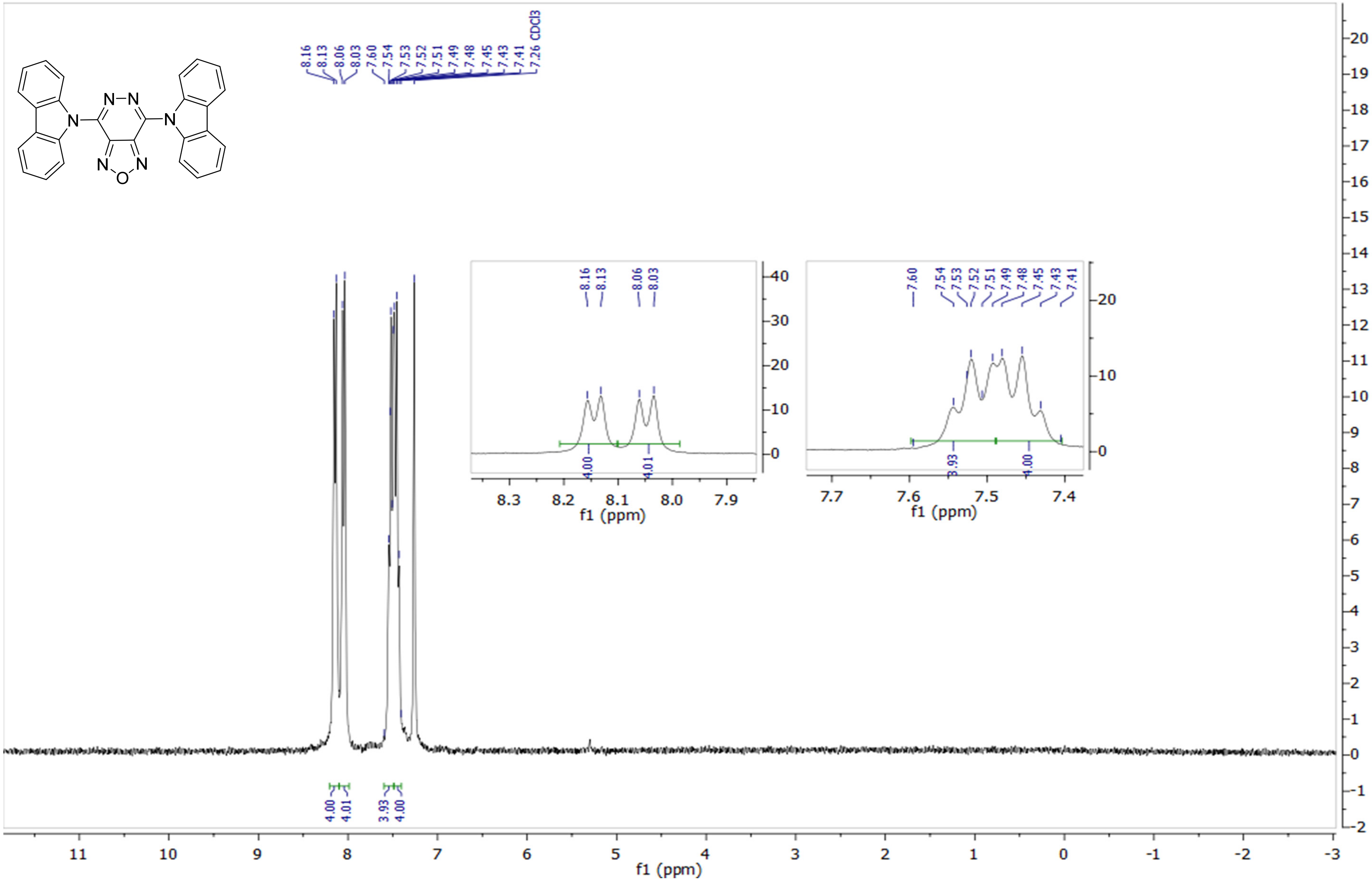

4,7-Bis(1,2,3,4,4a,9a-hexahydro-9H-carbazol-9-yl)-[1,2,5]oxadiazolo[3,4-d]pyridazine (4d) ^{13}C NMR(75 MHz)

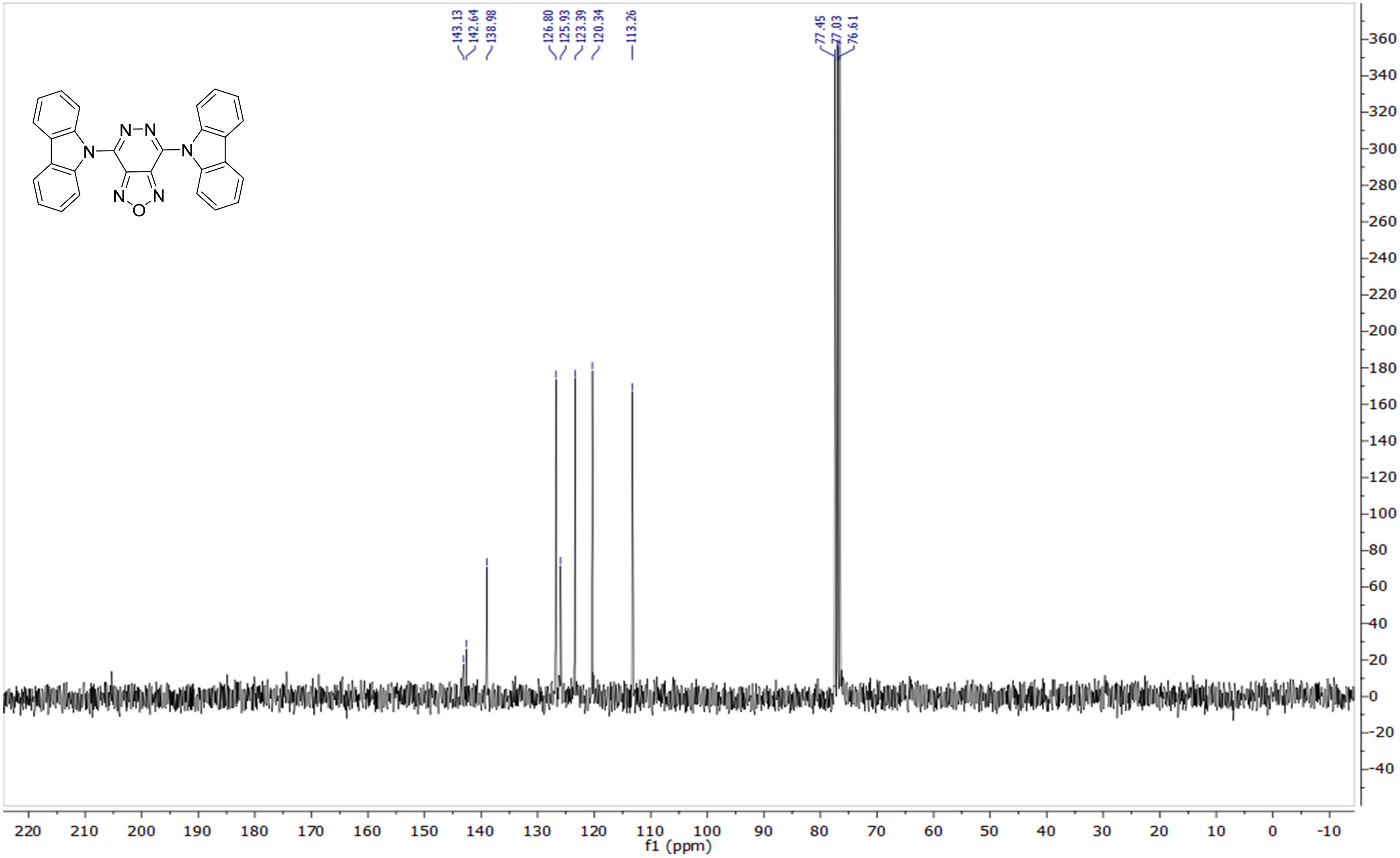

2. Single crystal X-ray diffraction data

X-ray crystallographic data and refinement details.

X-ray diffraction data for **3d** and **4d** were collected at 100K on a four-circle Rigaku XtaLAB Synergy-S diffractometer equipped with a HyPix6000HE area-detector (kappa geometry, shutterless ω-scan technique), using graphite monochromatized Cu K_{α}-radiation. The intensity data were integrated and corrected for absorption and decay by the CrysAlisPro program.[1] The structures were solved by dual methods using SHELXT[2] and refined by the full-matrix least-squares minimization method on F^2 using SHELXL-2018[3] in the OLEX2 program.[4] All non-hydrogen atoms were refined with individual anisotropic displacement parameters. All hydrogen atoms were placed in ideal calculated positions and refined as riding atoms with relative isotropic displacement parameters. The disorder in **3d** was handled in a regular way – distances of chemically equivalent bonds were restrained to be the equal within 0.02Å (SADI restrain of the SHELXL program), the atomic displacement parameters for equivalent atoms were set to be the same (EADP).

The Hirshfeld surface analysis was performed with the CrystalExplorer21 (version 21.5).[5] The *SHELXTL* program suite[6] and the *Mercury* program[7] were used for molecular graphics. Crystal data, data collection and structure refinement details are summarized in Table S1. The structures have been deposited at the Cambridge Crystallographic Data Center with the reference CCDC numbers 2401696 (**3d**) and 2401695 (**4d**); they also contain the supplementary crystallographic data. These data can be obtained free of charge from the CCDC *via* https://www.ccdc.cam.ac.uk/structures/ Geometrical data (bond distances, interatomic angles, dihedral angles and so on) are summarized in Tables S2-S5 for **3d** and Tables S6, S7 for **4d**.

Table S1. Crystal data and structure refinement for compound **4d** and **3d**.

Compound	**3d**•½$CHCl_3$	**4d**
Empirical formula	$C_{28}H_{16}N_6O_2$•0.5$CHCl_3$	$C_{28}H_{16}N_6O$
Formula weight	528.15	452.47
Temperature, K	99.9(3)	100.0(1)
Wavelength, Å	1.54184	1.54184
Crystal system	Orthorhombic	Orthorhombic
Space group	$Pca2_1$	*Pbca*
Unit cell dimensions:		
a, Å	19.21000(10)	19.3754(3)
b, Å	9.60270(10)	8.53240(10)
c, Å	25.2200(2)	25.1259(4)
Volume, $Å^3$	4652.28(7)	4153.78(10)
Z / Z′	8 / 2	8 / 1
Calculated density, $g\cdot cm^{-3}$	1.508	1.447
Absorption coefficient (μ), mm^{-1}	2.335	0.745
F(000)	2168	1872
Crystal size, mm	0.44 x 0.12 x 0.09	0.05 x 0.03 x 0.01
θ range, deg	3.505-77.857	3.518-79.789
Reflections		
Collected	33773	28598
Independent [R_{int}]	8793 [0.0267]	4443 [0.0732]
Observed with I>2σ(*I*)	8656	4039
Parameters / restraints / data	737 / 70 / 8793	316 / 0 / 4443
Goodness-of-fit on F^2	1.043	1.068
Completeness to θ_{full} / θ_{max}	1.000 / 0.988	0.997 / 0.985
R_1 / wR_2 for I>2σ(*I*)	0.0366 / 0.0950	0.0773 / 0.2042
R_1 / wR_2 (all reflections)	0.0372 / 0.0955	0.0804 / 0.2081
ρ_{max}/ρ_{min}, $e\cdot Å^{-3}$	0.484 / -0.582	0.503 / -0.458
Absolute structure parameter	0.430(14)*	-
CCDC number	2401696	2401695

* The crystallographic model of **3d** was refined as a racemic twin.

Crystal structure of 3d

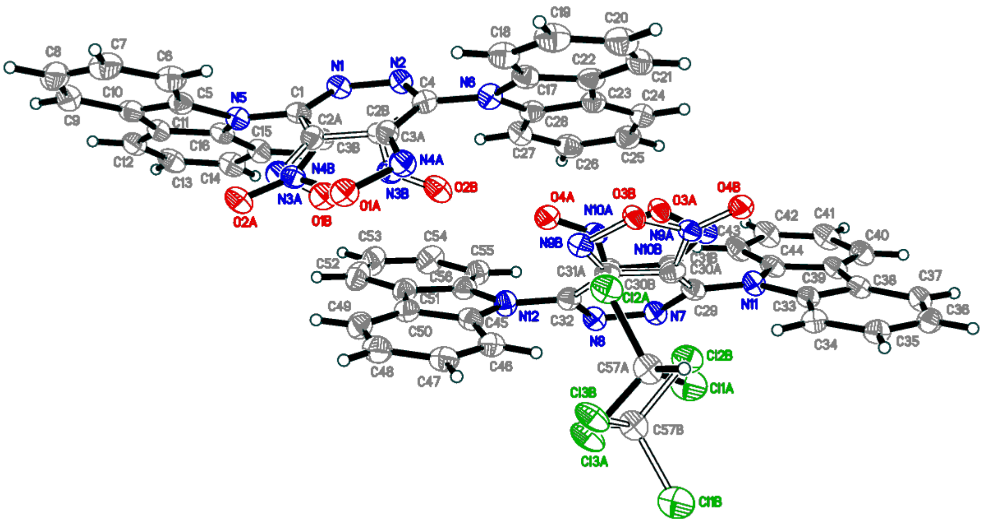

Figure S1. The structure of **3d**. Thermal ellipsoids are set to a 50% probability level.

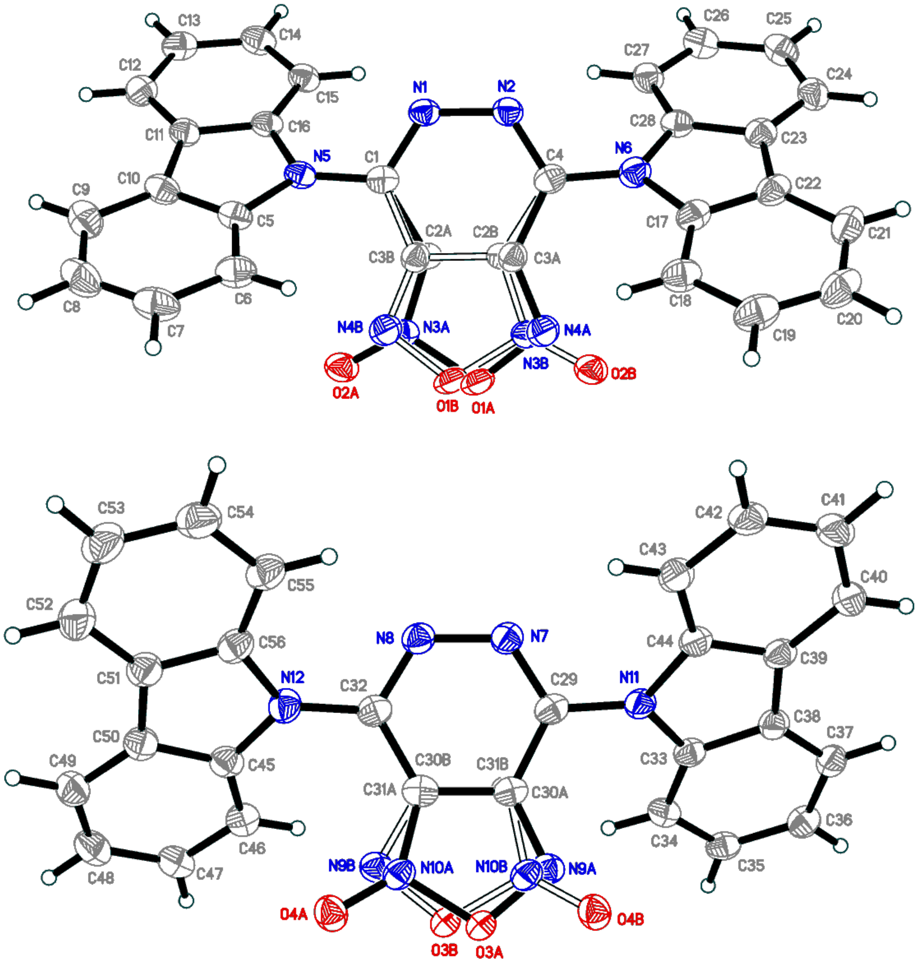

Figure S2. Two crystallographicaly independent molecules of **3d**. Thermal ellipsoids are set to a 50% probability level.

Compound **3d** crystalizes from chloroform as a racemic twin in a non-centrosymmetric orthorhombic space group (*Pca2₁*) with the domain ratio of 0.570(14):0.430(14). Its asymmetric unit (Fig. S1) contains two molecules of **3d** and a chloroform molecule. The latter is disordered over two positions with the occupancy ratio of 0.6593(17):0.3407(17). Both molecules of **3d** exhibit a disorder of the $C_2N_2O_2$ furoxan fragment (Fig. S2); the corresponding disorder ratios are 0.794(4): 0.206(4) for the first molecules (the top figure; atoms C2A, N3A, O1A, N4A, C3A, O2A / C2B, N3B, O1B, N4B, C3B, O2B) and 0.660(4):0.340(4) for the second molecule (the bottom figure; atoms C30A, N9A, O3A, N10A, C31A, O4A / C30B, N9B, O3B, N10B, C31B, O4B).

Table S2. Selected bond lengths (Å) for **3d**.

N1-N2	1.383(4)	C2B-C4	1.490(13)	C12-C13	1.380(5)
N1-C1	1.308(4)	C3B-C1	1.400(14)	C13-C14	1.399(5)
N2-C4	1.311(4)	N5-C1	1.390(4)	C14-C15	1.392(5)
N3A-O1A	1.466(6)	N5-C5	1.420(4)	C15-C16	1.390(4)
N3A-O2A	1.213(6)	N5-C16	1.410(4)	C17-C18	1.395(4)
N3A-C2A	1.342(8)	N6-C4	1.392(4)	C17-C22	1.401(5)
N4A-O1A	1.380(7)	N6-C17	1.410(4)	C18-C19	1.387(5)
N4A-C3A	1.309(9)	N6-C28	1.413(4)	C19-C20	1.393(6)
C2A-C3A	1.417(7)	C5-C6	1.393(4)	C20-C21	1.389(5)
C2A-C1	1.462(7)	C5-C10	1.397(5)	C21-C22	1.404(4)
C3A-C4	1.410(8)	C6-C7	1.388(5)	C22-C23	1.446(4)
N3B-O1B	1.474(14)	C7-C8	1.393(6)	C23-C24	1.398(4)
N3B-O2B	1.215(14)	C8-C9	1.384(5)	C23-C28	1.406(4)
N3B-C2B	1.364(17)	C9-C10	1.395(5)	C24-C25	1.382(5)
N4B-O1B	1.365(14)	C10-C11	1.450(4)	C25-C26	1.398(5)
N4B-C3B	1.308(18)	C11-C12	1.400(4)	C26-C27	1.386(5)
C2B-C3B	1.416(17)	C11-C16	1.402(4)	C27-C28	1.390(4)
N7-N8	1.379(4)	C30B-C32	1.44(2)	C40-C41	1.385(5)
N7-C29	1.307(4)	C31B-C29	1.44(2)	C41-C42	1.399(5)
N8-C32	1.314(4)	N11-C29	1.388(4)	C42-C43	1.388(5)
O3A-N9A	1.370(5)	N11-C33	1.416(4)	C43-C44	1.395(4)
O3A-N10A	1.483(5)	N11-C44	1.416(4)	C45-C46	1.391(4)
O4A-N10A	1.209(5)	N12-C32	1.387(4)	C45-C50	1.406(4)
N9A-C30A	1.327(8)	N12-C45	1.407(4)	C46-C47	1.384(5)
N10A-C31A	1.342(10)	N12-C56	1.418(4)	C47-C48	1.402(5)
C30A-C31A	1.402(7)	C33-C34	1.393(4)	C48-C49	1.377(5)
C30A-C29	1.435(6)	C33-C38	1.406(4)	C49-C50	1.398(5)

C31A-C32	1.428(7)	C34-C35	1.387(4)	C50-C51	1.454(4)
O3B-N9B	1.352(17)	C35-C36	1.395(5)	C51-C52	1.393(5)
O3B-N10B	1.453(19)	C36-C37	1.386(4)	C51-C56	1.401(5)
O4B-N10B	1.19(2)	C37-C38	1.405(4)	C52-C53	1.386(5)
N9B-C30B	1.33(3)	C38-C39	1.452(4)	C53-C54	1.394(6)
N10B-C31B	1.35(3)	C39-C40	1.391(4)	C54-C55	1.384(5)
C30B-C31B	1.41(2)	C39-C44	1.400(4)	C56-C55	1.390(5)

Table S3. Selected bond angles (°) in the crystals for compound **3d**.

C1-N1-N2	122.3(2)	C4-N6-C17	127.6(3)	C12-C13-C14	120.6(3)
C4-N2-N1	122.0(2)	C4-N6-C28	124.1(3)	C15-C14-C13	121.7(3)
O2A-N3A-O1A	118.0(5)	C17-N6-C28	108.3(3)	C16-C15-C14	117.1(3)
O2A-N3A-C2A	136.7(5)	N1-C1-C2A	116.9(4)	C11-C16-N5	108.4(2)
C2A-N3A-O1A	105.3(5)	N1-C1-C3B	124.3(7)	C15-C16-N5	129.4(3)
C3A-N4A-O1A	106.0(5)	N1-C1-N5	118.7(2)	C15-C16-C11	122.1(3)
N4A-O1A-N3A	108.9(5)	N5-C1-C2A	124.3(4)	C18-C17-N6	129.5(3)
N3A-C2A-C3A	107.2(5)	N5-C1-C3B	117.1(7)	C18-C17-C22	121.9(3)
N3A-C2A-C1	131.1(6)	N2-C4-C3A	122.2(4)	C22-C17-N6	108.5(3)
C3A-C2A-C1	121.5(6)	N2-C4-C2B	115.3(7)	C19-C18-C17	117.2(3)
N4A-C3A-C2A	112.6(6)	N2-C4-N6	117.6(3)	C18-C19-C20	121.9(3)
N4A-C3A-C4	132.1(6)	N6-C4-C3A	120.0(4)	C21-C20-C19	120.8(3)
C4-C3A-C2A	114.9(6)	N6-C4-C2B	126.9(7)	C20-C21-C22	118.5(3)
O2B-N3B-O1B	119.4(9)	C6-C5-N5	129.5(3)	C17-C22-C21	119.7(3)
O2B-N3B-C2B	136.4(12)	C6-C5-C10	122.0(3)	C17-C22-C23	107.5(3)
C2B-N3B-O1B	104.2(11)	C10-C5-N5	108.4(3)	C21-C22-C23	132.8(3)
C3B-N4B-O1B	105.2(13)	C7-C6-C5	116.8(3)	C24-C23-C22	132.9(3)
N4B-O1B-N3B	110.3(12)	C6-C7-C8	121.9(3)	C24-C23-C28	119.6(3)
N3B-C2B-C3B	106.2(11)	C9-C8-C7	120.8(3)	C28-C23-C22	107.5(3)
N3B-C2B-C4	129.5(13)	C8-C9-C10	118.5(3)	C25-C24-C23	118.5(3)
C3B-C2B-C4	123.6(12)	C5-C10-C11	107.5(3)	C24-C25-C26	120.9(3)
N4B-C3B-C2B	113.9(12)	C9-C10-C5	120.0(3)	C27-C26-C25	121.8(3)
N4B-C3B-C1	132.4(14)	C9-C10-C11	132.5(3)	C26-C27-C28	117.0(3)
C1-C3B-C2B	112.4(12)	C12-C11-C10	132.9(3)	C23-C28-N6	108.2(3)
C1-N5-C5	126.6(2)	C12-C11-C16	119.6(3)	C27-C28-N6	129.5(3)
C1-N5-C16	124.9(2)	C16-C11-C10	107.6(3)	C27-C28-C23	122.2(3)
C16-N5-C5	108.2(2)	C13-C12-C11	118.9(3)		
C29-N7-N8	122.0(2)	N9B-C30B-C31B	122(2)	C40-C41-C42	120.8(3)
C32-N8-N7	121.7(2)	N9B-C30B-C32	120(3)	C43-C42-C41	121.3(3)
C29-N11-C33	126.8(2)	C31B-C30B-C32	118(3)	C42-C43-C44	117.2(3)

C29-N11-C44	125.2(2)	N10B-C31B-C30B	98(2)	C39-C44-N11	109.0(2)
C44-N11-C33	107.9(2)	N10B-C31B-C29	138(4)	C43-C44-N11	128.9(3)
C32-N12-C45	126.0(3)	C30B-C31B-C29	118(3)	C43-C44-C39	122.1(3)
C32-N12-C56	125.5(3)	N11-C29-C30A	120.5(5)	C46-C45-N12	129.1(3)
C45-N12-C56	108.2(2)	N11-C29-C31B	121.1(18)	C46-C45-C50	122.1(3)
N7-C29-C30A	120.4(5)	N8-C32-C31A	119.2(5)	C50-C45-N12	108.7(3)
N7-C29-C31B	120.1(18)	N8-C32-C30B	120.0(17)	C47-C46-C45	117.4(3)
N7-C29-N11	118.8(3)	N8-C32-N12	118.4(3)	C46-C47-C48	121.3(3)
N9A-O3A-N10A	108.4(3)	N12-C32-C31A	122.3(5)	C49-C48-C47	120.9(3)
C30A-N9A-O3A	104.7(4)	N12-C32-C30B	121.5(17)	C48-C49-C50	119.1(3)
O4A-N10A-O3A	118.3(4)	C34-C33-N11	129.2(3)	C45-C50-C51	107.2(3)
O4A-N10A-C31A	135.0(6)	C34-C33-C38	122.3(3)	C49-C50-C45	119.2(3)
C31A-N10A-O3A	106.7(5)	C38-C33-N11	108.5(3)	C49-C50-C51	133.5(3)
N9A-C30A-C31A	115.0(5)	C35-C34-C33	117.1(3)	C52-C51-C50	132.8(3)
N9A-C30A-C29	127.8(7)	C34-C35-C36	121.7(3)	C52-C51-C56	119.7(3)
C31A-C30A-C29	117.0(8)	C37-C36-C35	120.9(3)	C56-C51-C50	107.4(3)
N10A-C31A-C30A	105.0(7)	C36-C37-C38	118.7(3)	C53-C52-C51	118.2(3)
N10A-C31A-C32	134.5(9)	C33-C38-C39	107.5(3)	C52-C53-C54	121.1(3)
C30A-C31A-C32	119.8(8)	C37-C38-C33	119.2(3)	C55-C54-C53	121.9(3)
N9B-O3B-N10B	111.4(14)	C37-C38-C39	133.3(3)	C51-C56-N12	108.5(3)
C30B-N9B-O3B	98.9(19)	C40-C39-C38	133.3(3)	C55-C56-N12	129.0(3)
O4B-N10B-O3B	120.7(18)	C40-C39-C44	119.6(3)	C55-C56-C51	122.6(3)
O4B-N10B-C31B	130(3)	C44-C39-C38	107.2(3)	C54-C55-C56	116.6(3)
C31B-N10B-O3B	109(2)	C41-C40-C39	119.0(3)		

The furoxan fragments of both molecules are not quite planar – some atoms have large deviations from the least-squares planes $N_4C_4O_2$, and the root mean square deviations of atomic positions (RMSD) are rather high (Table S4; planes 1-4). Moreover, each fragment exhibits slight folding along lines passing through atoms C1/C4 and C2A/C3A (or C2B/C3B) for the 1st molecule, and through atoms C29/C32 and C30A/C31A (or C30B/C31B) for the 2nd molecule; the estimated folding (dihedral) angles between flat fragments (N_2C_2/C_4 and $C_4/C_2N_2O_2$) are small (see Table S5). However, the disorder of the $C_4N_2O_2$ fragment for both crystallographically unique molecules does not allow to find the angles with reasonably small estimated standard deviations (ESDs). We may only undoubtedly state that π-conjugation within the $N_4C_4O_2$ heterocyclic π-system retains. We suppose that such double folding of the $N_4C_4O_2$ heterocycle should be a result of some electronic effects rather than induced by non-covalent contacts.

Table S4. Atom deviations (Å) and RMSD (Å) in from the best-fitted least-squares planes in **3d**.

Plane #	Atoms defining a plane / their deviations from the plane										RMSD
1	N1	N2	C1	C2A	C3A	C4	N3A	N4A	O1A	O2A	
	-0.026(3)	-0.072(3)	0.065(3)	0.075(7)	0.067(9)	-0.018(3)	-0.007(5)	0.004(5)	-0.024(6)	-0.064(3)	0.050
2	N1	N2	C1	C2B	C3B	C4	N3B	N4B	O1B	O2B	
	-0.098(4)	-0.037(4)	-0.043(6)	0.127(17)	0.108(16)	0.087(5)	-0.016(10)	0.000(11)	-0.031(13)	-0.097(7)	0.077
3	N7	N8	C29	C30A	C31A	C32	N9A	N10A	O3A	O4A	
	0.096(4)	0.024(6)	0.022(4)	-0.066(9)	-0.11(3)	-0.079(6)	-0.010(5)	0.016(7)	0.017(6)	0.091(7)	0.065
4	N7	N8	C29	C30B	C31B	C32	N9B	N10B	O3B	O4B	
	0.058(12)	0.08(2)	-0.035(11)	-0.10(13)	-0.22(5)	0.05(3)	0.00(4)	0.01(3)	0.05(3)	0.118(15)	0.095
5	C1	N1	N2	C4							
	-0.0008(8)	0.0015(15)	-0.0015(15)	0.0008(8)							0.0012
6	C1	C2A	C3A	C4							
	0.007(2)	-0.014(5)	0.013(5)	-0.007(2)							0.011
7	C2A	N3A	O1A	N4A	C3A	O2A					
	0.013(5)	0.003(5)	0.017(5)	-0.011(5)	-0.008(6)	-0.013(3)					0.012
8	C1	C2B	C3B	C4							
	-0.009(5)	-0.018(10)	0.018(9)	0.009(5)							0.014
9	C2B	N3B	O1B	N4B	C3B	O2B					
	0.016(10)	-0.023(10)	0.022(10)	-0.013(10)	-0.002(10)	-0.056(20)					0.017

Table S4 (*cont.*). Atom deviations (Å) and RMSD (Å) in from the best-fitted least-squares planes in **3d**.

	Atoms defining a plane / their deviations from the plane						RMS
	C29	N7	N8	C32			
	-0.0045(8)	0.0090(15)	-0.0090(15)	0.0045(8)			0.0071
	C29	C30A	C31A	C32			
	0.001(6)	-0.003(12)	0.003(13)	-0.001(7)			0.002
	C30A	N9A	O3A	N10A	C31A	O4A	
	0.072(13)	0.082(5)	-0.035(8)	-0.073(6)	-0.078(16)	0.033(2)	0.065
	C29	C30B	C31B	C32			
	0.02(3)	0.03(5)	-0.03(5)	-0.02(3)			0.026
	C30B	N9B	O3B	N10B	C31B	O4B	
	0.021(6)	-0.09(5)	-0.07(3)	0.081(19)	0.01(5)	0.049(11)	0.062

Each N-carbazolyl fragment ($C_{12}H_8N$) is slightly distorted from a plane – the corresponding RSMDs lie in the range from 0.012Å to 0.044Å. The N_2C_4-$C_{12}H_8N$ rotation (dihedral) angles (Table 5) are in the range of 41.6(1.5)-46.32(13)°, pointing out to a substantial loss of π-π conjugation between two heterocyclic fragments.

Table S5. Selected dihedral angles in **3d** (°).

Plane 1	Plane 2	angle
C1 N1 N2 C4	C1 C2A C3A C4	1.5(5)
C1 N1 N2 C4	C1 C2B C3B C4	0.4(9)
C29 N7 N8 C32	C29 C30A C31A C32	1.8(1.5)
C29 N7 N8 C32	C29 C30B C31B C32	5(6)
C1 C2A C3A C4	C2A N3A O1A N4A C3A O2A	5.6(6)
C1 C2B C3B C4	C2B N3B O1B N4B C3B O2B	9.4(1.3)
C29 C30A C31A C32	C30A N9A O3A N10A C31A O4A	9.4(1.9)
C29 C30B C31B C32	C30B N9B O3B N10B C31B O4B	14(5)
C2A N3A O1A C3A O2A	N1 N2 C1 C2A C3A C4	6.3(5)
C2B N3B O1B C3B O2B	N1 N2 C1 C2B C3B C4	9.4(1.1)
C30A N9A O3A N10A C31A O2A	N7 N8 C29 C30A C31A C32	10.2(1.3)
C30B N9B O3B N10B C31B O2B	N7 N8 C29 C30B C31B C32	13(5)
C2A N3A O1A N4A C3A O2A	C2B N3B O1B N4B C3B O2B	5.2(1.1)
C30A N9A O3A N10A C31A O4A	C30B N9B O3B N10B C31B O4B	4.1(8)
N1 N2 C1 C2A C3A C4	N5 C5..C16	46.21(13)
N1 N2 C1 C2A C3A C4	N6 C17..C28	46.32(13)
N1 N2 C1 C2B C3B C4	N5 C5..C16	45.4(3)
N1 N2 C1 C2B C3B C4	N6 C17..C28	45.5(2)
N7 N8 C29 C30A C31A C32	N11 C33..C44	45.3(4)
N7 N8 C29 C30A C31A C32	N12 C45..C56	45.0(4)
N7 N8 C29 C30B C31B C32	N11 C33..C44	41.9(1.5)
N7 N8 C29 C30B C31B C32	N12 C45..C56	41.6(1.5)

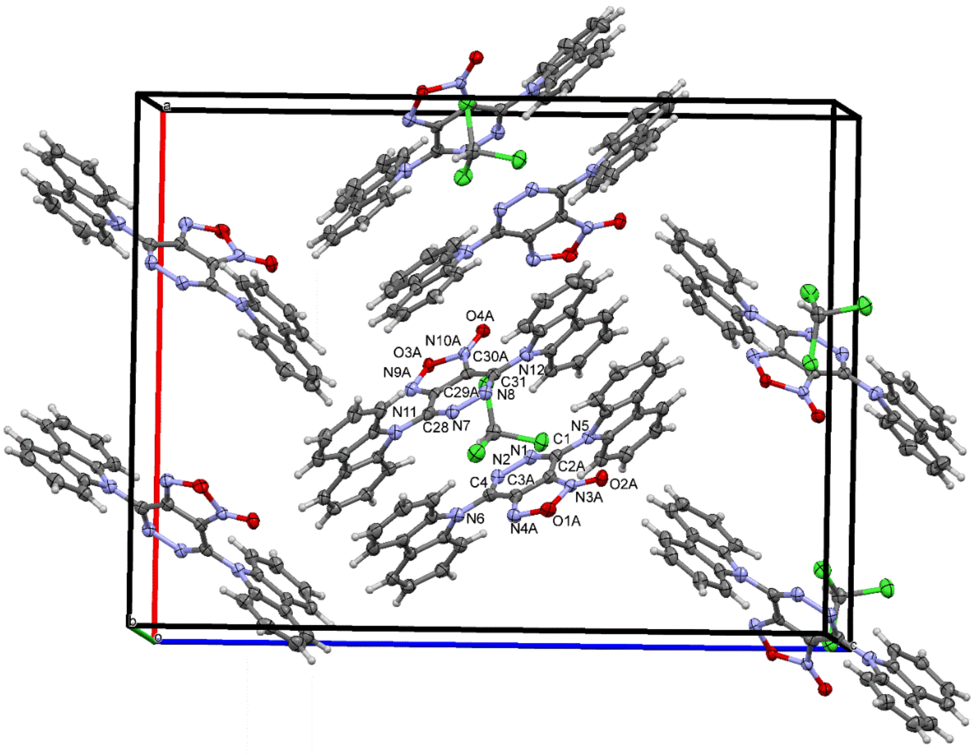

Figure S3. The crystal packing of **3d**. The disorder of the furoxan system is omitted. Thermal ellipsoids are set to a 50% probability level.

Hirschfeld surface analysis of **3d**•½$CHCl_3$ (Fig. S4) has revealed short contacts of chlorine atoms of the lattice chloroform molecule with oxygen atoms (11.6% of the total surface area) and with nitrogen atoms (8.7%) of the furoxan ring of **3d**; the chlorine atom is oriented toward the O3A-N10A-O4A triangle, where atom O4A bears a negative charge. This may indicate the presence of a weak halogen bond with an average length of 3.1 Å (distances Cl2A..O3A, Cl2A..N10A and Cl2A..O4A are 3.140, 3.193 and 3.026Å, correspondingly), and with location of a σ-hole at the chlorine atom. All chlorine atoms (Cl1A, Cl2A and Cl3A) also form weak hydrogen bonds Cl···H-C_{Aryl}. The chlorine atom Cl2B of the minor position of the crystallization chloroform molecule (site occupancy of 34.1%) is not directed toward either a possible formation of a halogen bond or a possible formation of a weak hydrogen bond with the aromatic system of **3d**. Positions of two other chlorine atoms do not change much, retaining weak hydrogen bonds Cl···H-C_{Aryl}. Although the formation of the halogen bond (the major chloroform position with the 65.9% site occupancy) leads to stabilization of the prevailing orientation of the chloroform molecule in the lattice, but it is a less significant factor for the stabilization of the molecule in the lattice than weak hydrogen bonds with aromatic fragments.

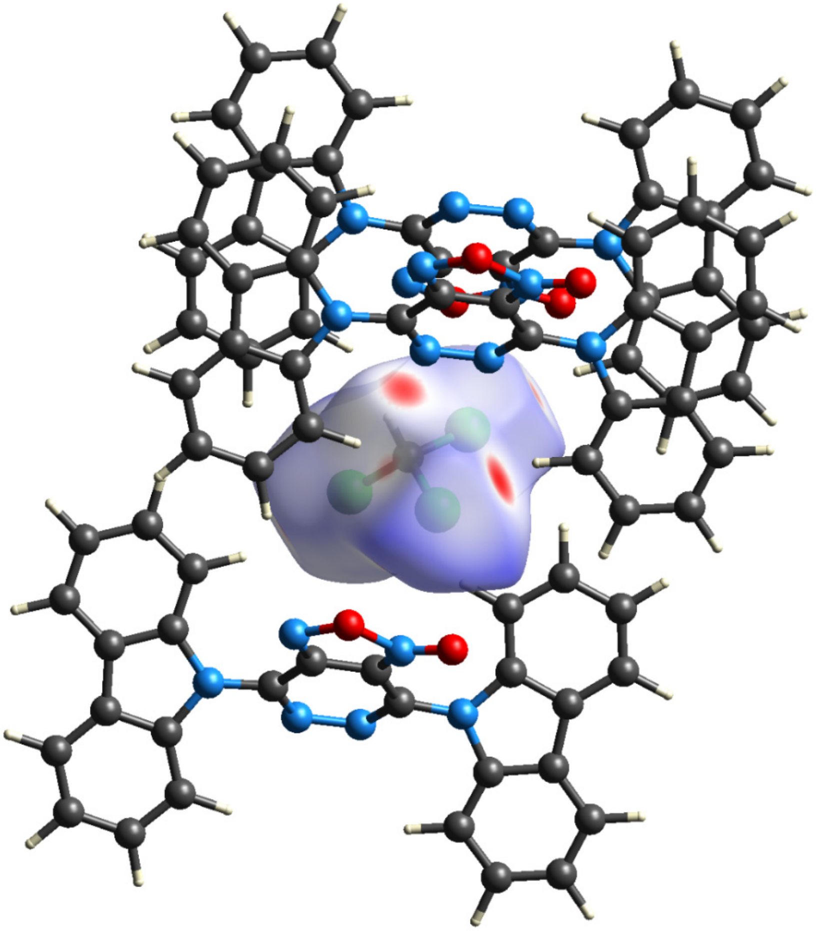

Figure S4. The Hirshfeld surface for a chloroform molecule and the interaction of the latter with molecules of **3d**.

Crystal structure of 4d

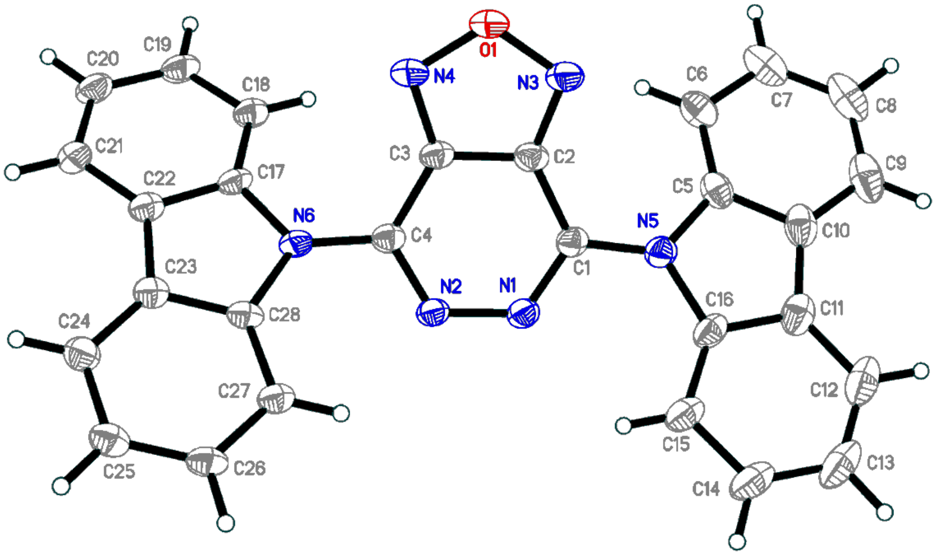

Figure S5. The molecular structure of **4d**. Thermal ellipsoids are set to a 50% probability level.

The oxadiazolopyridazine heterocyclic system N_4C_4O in **4d** is also not planar. The RSMD for all nine atoms is 0.069 Å; the out-of-plane deviations are rather high (with the exception of N4 – 0.006(13)Å) and vary for eight remaining atoms from 0.0357(13)Å for C4 to 0.0955(16)Å for C3. The dihedral angle for planes defined by atoms C2, C3, N3, N4, O1 (RSMD = 0.005Å). and by C1..C4, N1, N2 (RSMD = 0.027Å) is 9.02(12)°. The N_4C_4O fragment is also bent over two lines going through atoms C1, C4 and atoms C2, C3. The corresponding dihedral angles are also small: 5.08(16)° for planes defined by atoms C1, N1, N2, C4 (RSMD = 0.007Å) and C1..C4 (RSMD = 0.011Å), and 6.75(15)° for planes C1..C4 and C2, C3, N3, N4, O1 (RSMD = 0.005Å). The dihedral angle for planes C2, C3, N3, N4, O1 and N1, N2, C1..C4 (RSMD = 0.027Å) is 9.02(12)°. These dihedral angles are rather small, therefore π-conjugation within the N_4C_4O heterocyclic π-system retains. In case of **4d**, there are no short non-covalent contacts with the furazan heterocycle, therefore the folding of the oxadiazolopyridazine heterocyclic system is likely the result of some electronic effects. The presence of intramolecular van der Waals interactions between the heterocycles in **4d** also leads to a substantial loss of π-π conjugation between the oxadiazolopyridazine heterocycle and two N-carbazolyl fragments but to a lesser extent than for **3d**: the dihedral angles between planes C1..C4, N1, N2 / N5, C5..C16 (RSMD = 0.024Å) and planes C1..C4, N1, N2 / N6, C17..C28 (RSMD = 0.075Å) are 29.38(6)° and 40.02(5)°, correspondingly. The first lower value is due to the presence of short contacts of atoms N1 and N2 of the pyridazine cycle with atoms of N-carbazolyl substituents of an adjacent molecule (Fig. S6); corresponding vales are 3.024 Å for N2···C1, 3.080 Å for N2···N5, 3.248 Å for N2···C16,

2.959 Å for N1···N5, 3.061 Å for N1···C16, and 3.241 Å for N1···C5. These contacts form infinite 1D chains along the *b* direction (Fig. S7) and should facilitate intermolecular charge transfer between neighboring molecules of **4d**.

Table S6. Selected bond lengths (Å) for **4d**.

O1-N3	1.381(2)	C2-C3	1.413(3)	C15-C16	1.396(3)
O1-N4	1.373(2)	C3-C4	1.443(2)	C17-C18	1.393(2)
N1-N2	1.374(2)	C5-C6	1.400(3)	C17-C22	1.404(3)
N1-C1	1.311(2)	C5-C10	1.406(3)	C18-C19	1.388(3)
N2-C4	1.308(2)	C6-C7	1.391(3)	C19-C20	1.395(3)
N3-C2	1.320(2)	C7-C8	1.392(4)	C20-C21	1.386(3)
N4-C3	1.318(2)	C8-C9	1.391(4)	C21-C22	1.403(2)
N5-C1	1.382(2)	C9-C10	1.394(3)	C22-C23	1.449(2)
N5-C5	1.413(2)	C10-C11	1.447(3)	C23-C24	1.396(3)
N5-C16	1.425(2)	C11-C12	1.400(3)	C23-C28	1.401(3)
N6-C4	1.383(2)	C11-C16	1.404(3)	C24-C25	1.389(3)
N6-C17	1.413(2)	C12-C13	1.380(4)	C25-C26	1.396(3)
N6-C28	1.418(2)	C13-C14	1.391(4)	C26-C27	1.392(3)
C1-C2	1.450(2)	C14-C15	1.397(3)	C27-C28	1.396(2)

Table S7. Selected bond angles (°) in the crystals for compound **4d**.

N4-O1-N3	113.06(13)	N2-C4-C3	119.03(16)	C15-C16-C11	121.96(18)
C1-N1-N2	123.09(15)	N6-C4-C3	122.75(15)	C18-C17-N6	129.23(17)
C4-N2-N1	122.15(15)	C6-C5-N5	130.21(18)	C18-C17-C22	121.87(17)
C2-N3-O1	103.98(15)	C6-C5-C10	121.01(18)	C22-C17-N6	108.61(15)
C3-N4-O1	103.78(15)	C10-C5-N5	108.70(18)	C19-C18-C17	117.20(18)
C1-N5-C5	127.75(16)	C7-C6-C5	117.5(2)	C18-C19-C20	121.89(18)
C1-N5-C16	124.12(16)	C6-C7-C8	121.9(2)	C21-C20-C19	120.66(17)
C5-N5-C16	108.09(16)	C9-C8-C7	120.2(2)	C20-C21-C22	118.60(18)
C4-N6-C17	126.64(15)	C8-C9-C10	119.0(2)	C17-C22-C23	107.34(15)
C4-N6-C28	125.44(14)	C5-C10-C11	107.21(17)	C21-C22-C17	119.68(17)
C17-N6-C28	107.91(14)	C9-C10-C5	120.1(2)	C21-C22-C23	132.83(17)
N1-C1-N5	117.18(15)	C9-C10-C11	132.6(2)	C24-C23-C22	132.46(17)
N1-C1-C2	118.63(16)	C12-C11-C10	132.1(2)	C24-C23-C28	119.99(17)
N5-C1-C2	124.12(16)	C12-C11-C16	119.9(2)	C28-C23-C22	107.49(15)
N3-C2-C1	132.61(17)	C16-C11-C10	108.01(17)	C25-C24-C23	118.48(17)
N3-C2-C3	109.16(16)	C13-C12-C11	118.7(2)	C24-C25-C26	120.82(17)
C3-C2-C1	117.89(15)	C12-C13-C14	120.8(2)	C27-C26-C25	121.79(17)

N4-C3-C2	109.99(16)	C13-C14-C15	122.1(2)	C26-C27-C28	116.82(17)
N4-C3-C4	130.74(17)	C16-C15-C14	116.6(2)	C23-C28-N6	108.50(15)
C2-C3-C4	118.75(15)	C11-C16-N5	107.96(17)	C27-C28-N6	129.40(17)
N2-C4-N6	117.94(15)	C15-C16-N5	130.04(18)	C27-C28-C23	122.09(17)

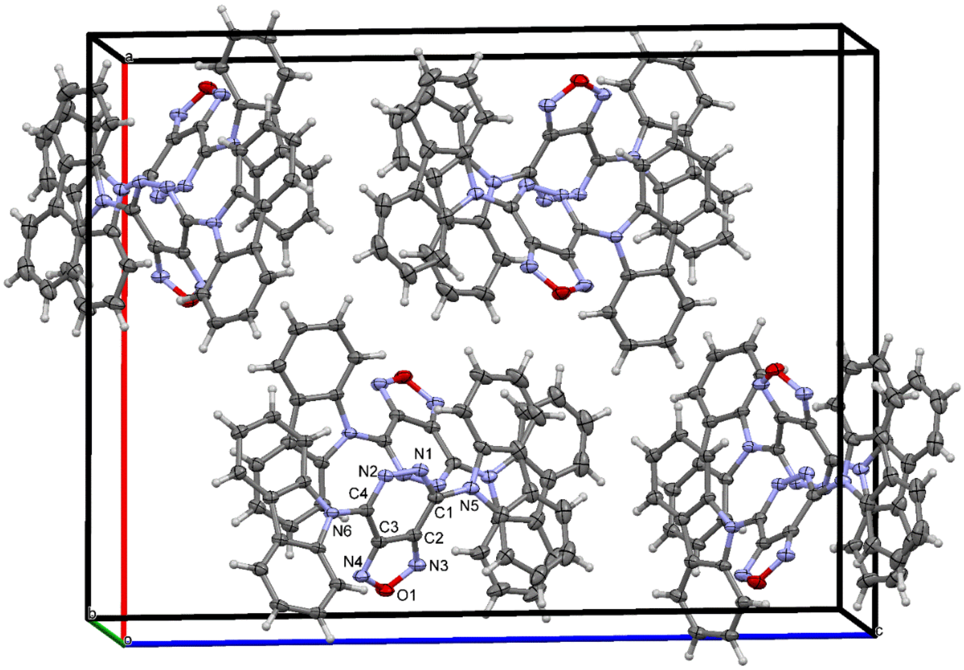

Figure S6. The packing plot of the unit cell in **4d**. Thermal ellipsoids are set to a 50% probability level.

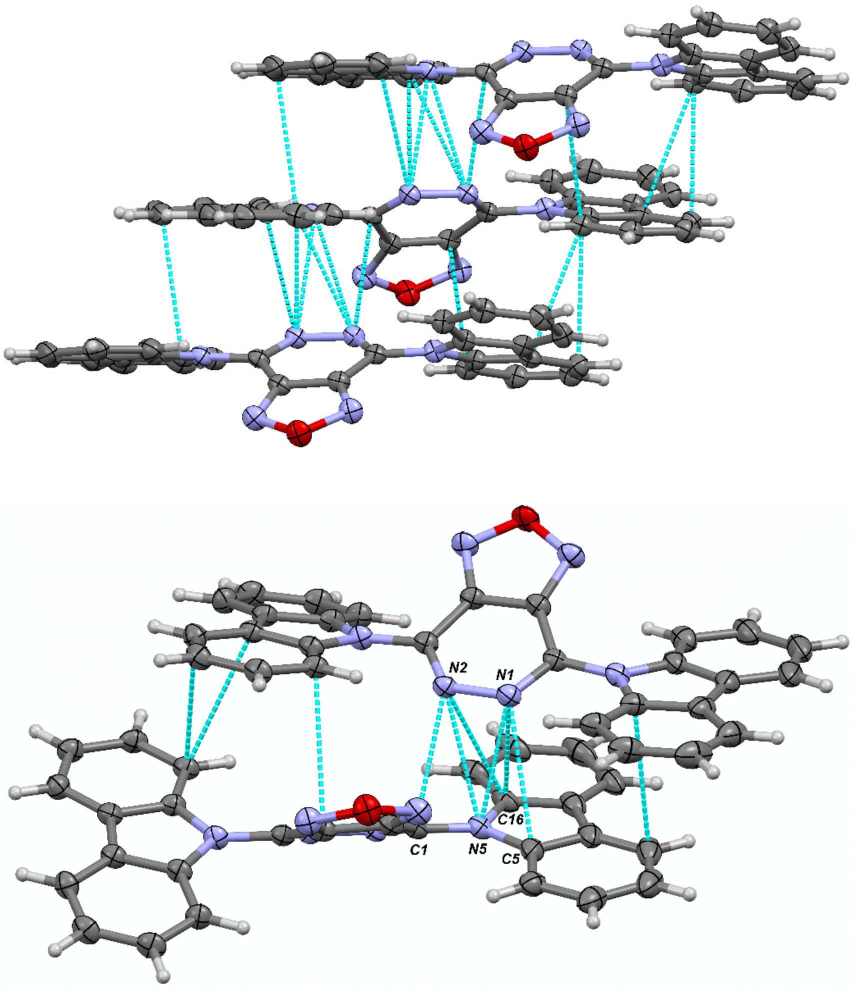

Figure S7. Intermolecular non-valent contacts in the crystal of **4d**. Thermal ellipsoids are set to a 50% probability level.

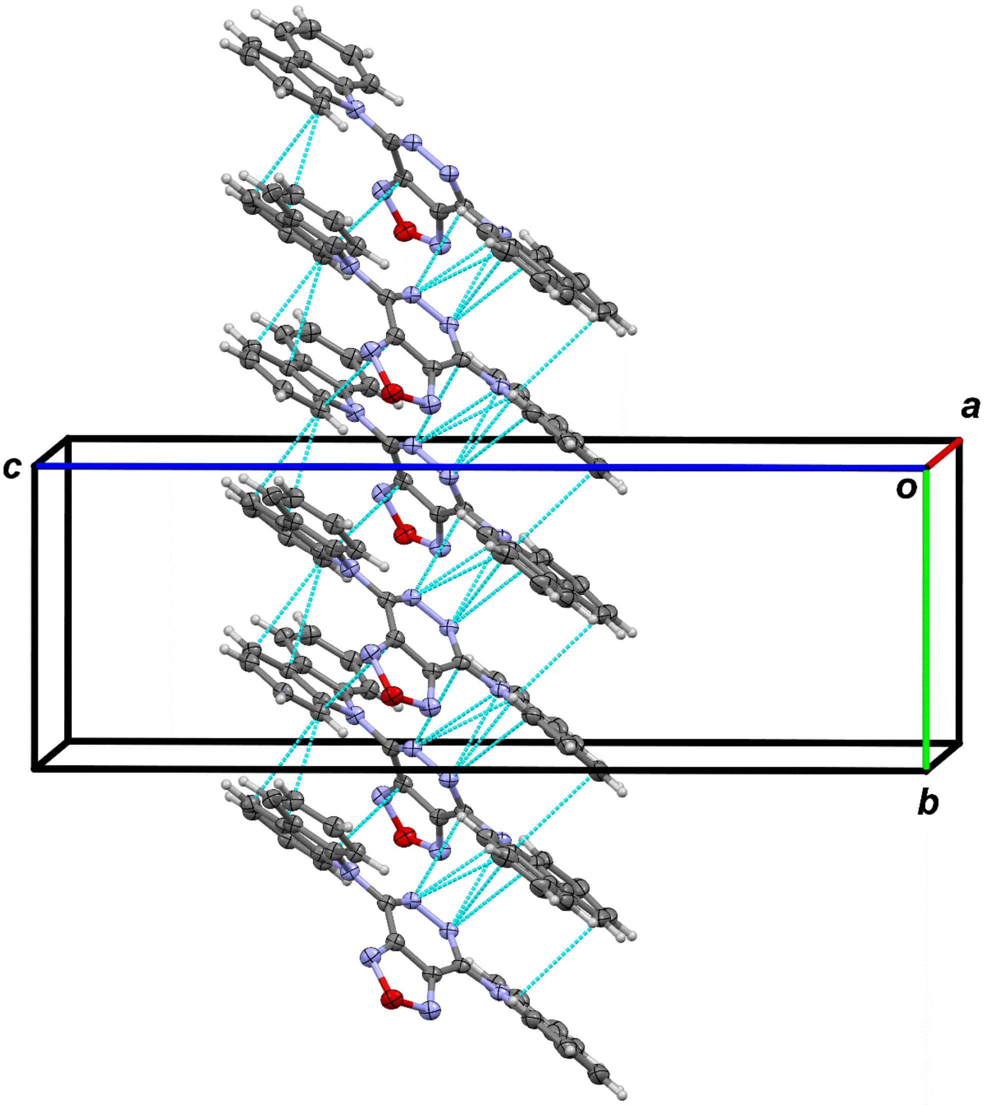

Figure S8. A 1D chain of molecules formed by intermolecular non-valent contacts along the *b* direction in the crystal of **4d**. Thermal ellipsoids are set to a 50% probability level.

The Hirshfeld surface analysis for **4d** (Fig. S9) confirms the presence of N···N (2.2% of overall surface area) and N···C (6.2%) short contacts in the heterocycle area. It might be noted that C···C π-π-interactions take 9.0% of the Hirshfeld surface area.

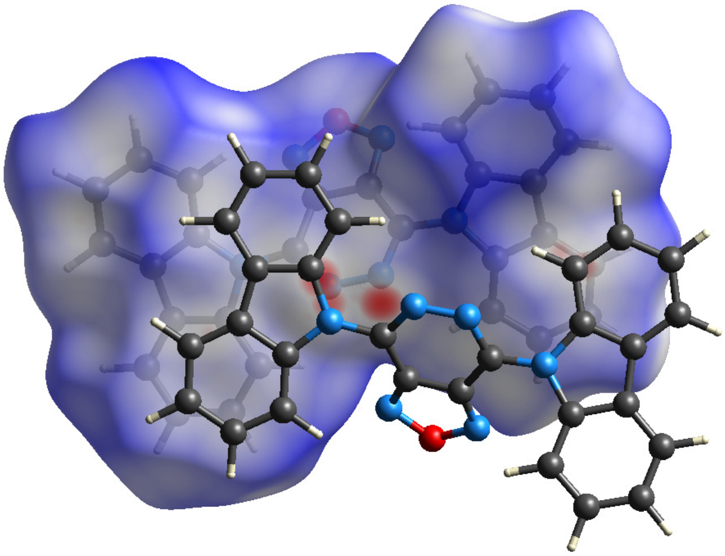

Figure S9. The Hirshfeld surface for **4d**.

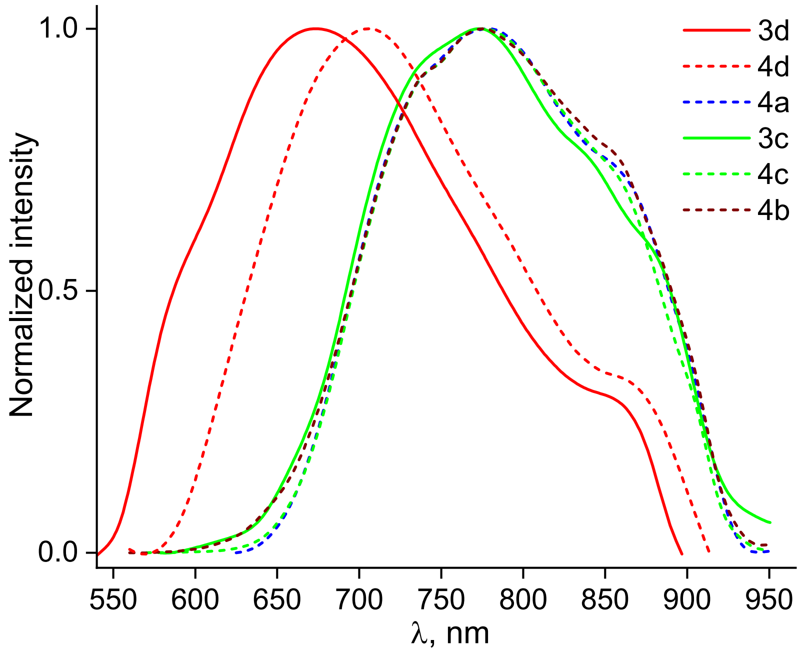

Figure S10. Normalized PL spectra recorded for all dyes dissolved in THF upon photoexcitation at λ_{abs} of the ICT state.

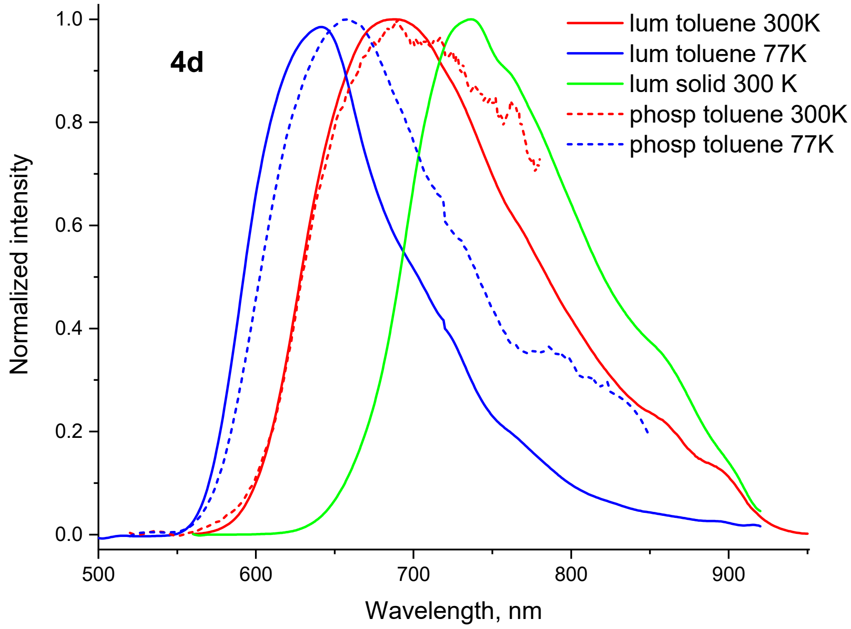

Figure S11. Normalized luminescence and phosphorescence spectra (time delay 5 us) for the toluene dissolved **4d** dye at 300 K and 77 K; green curve is the luminescence spectrum for crystalline powder **4d** dye. The excitation wavelength is 450 nm.

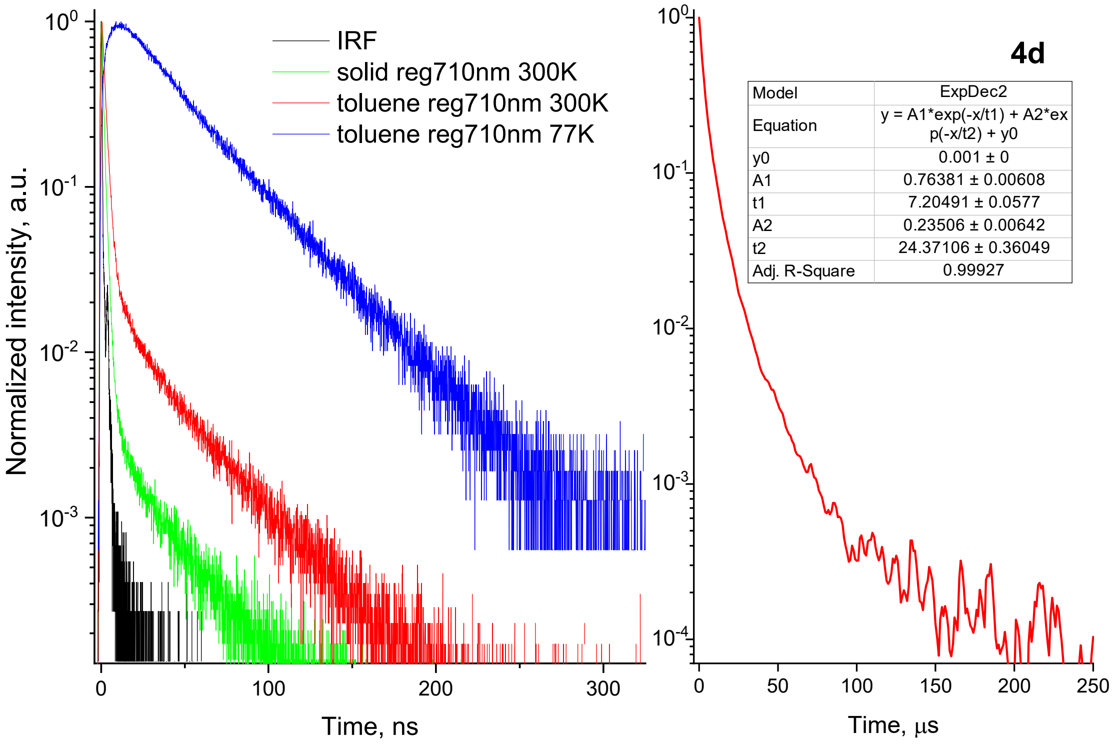

Figure S12. Left: Normalized PL decays for the toluene dissolved **4d** dye at 300 K and 77 K measured by TCSPC method; green curve is the PL decay for crystalline powder **4d** dye; **Right:** Phosphorescence decay for toluene dissolved **4d** dye measured by the Single-Shot Transient Digitizer (SSTD); the laser excitation wavelength is 500 nm.

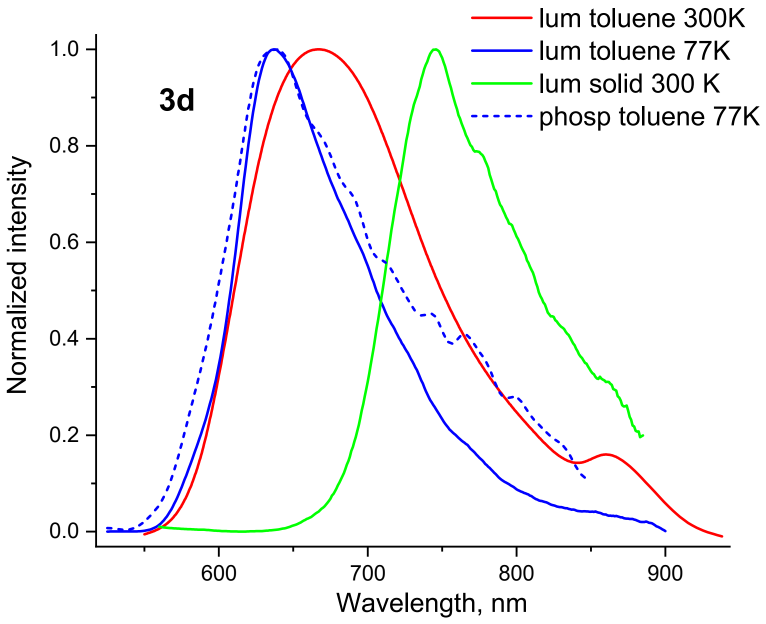

Figure S13. Normalized luminescence and phosphorescence spectra (time delay 5 us) for the toluene dissolved **3d** dye at 300 K and 77 K; green curve is the luminescence spectrum for crystalline powder **3d** dye. The excitation wavelength is 450 nm.

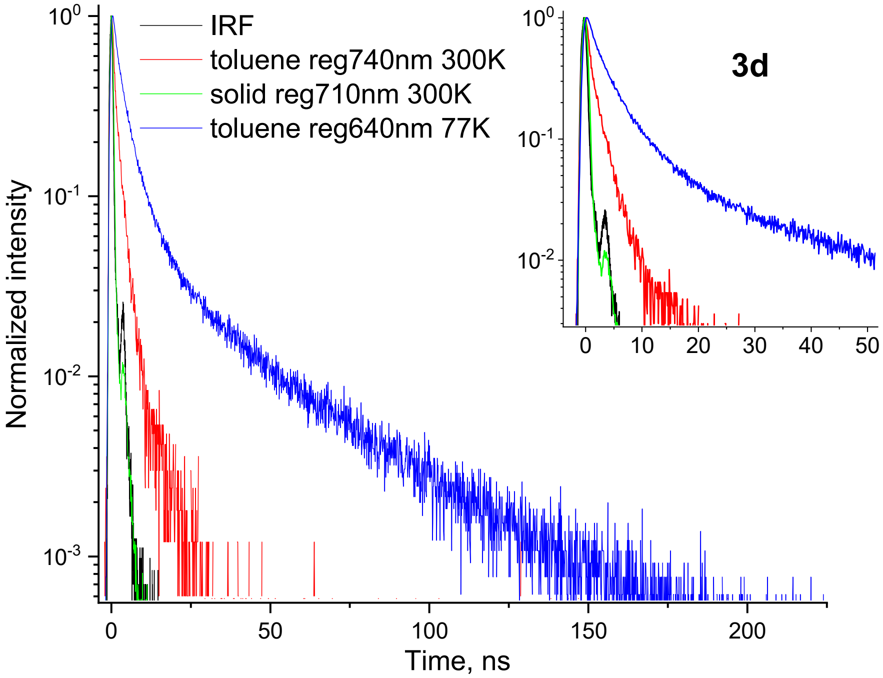

Figure S14. Normalized PL decays for the toluene dissolved **3d** dye at 300 K and 77 K; green curve is the PL decay for crystalline powder **3d** dye; The laser excitation wavelength is 450 nm

4. Quantum-chemical calculations

DFT calculations were performed using the functional PBE1PBE(PBE0)[8] and the DEF2TZVP[9] basis with the GD3BJ empirical dispersion in the Gaussian 2016 program[10] (gas phase, 298K).

Geometry of each structure (**3d** and **4d**) was calculated twice, using different the initial atom coordinates, that (1) were directly taken from the crystallographic data, (2) were found by consequent geometry optimization with molecular mechanics (MM+) and semi-empirical calculations (PM3). Surprisingly, both calculations were converged to the same geometry in each case, but converging from crystallographic coordinates took approximately two times longer.

Table S8. Summary for **4a** (tautomer **A**), **5b, 6a** and **8**. Positions of non-hydrogen atoms were taken from crystallographic data and fixed; positions of hydrogen atoms were optimized.

	**3d**	**4b**
Calculation Type	FOPT	FREQ
Calculation Method	RPBE1PBE	RPBE1PBE
Basis Set	def2TZVP	def2TZVP
Charge	0	0
Spin	Singlet	Singlet
Solvation	None	None
Dipole Moment, Debye	0.47778646	0.52096042
Point Group	C1	C1
Electronic Energy (EE),Hartree	-1554.2781	-1479.1547
HOMO energy, eV	-0.22604	-0.22565
LUMO energy, eV	-0.12062	-0.12018

Resulting geometries

The optimized geometry of **3d**.

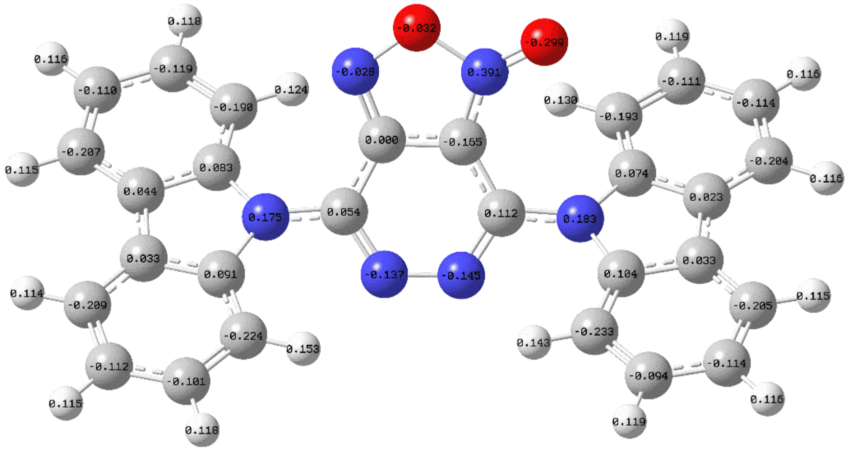

Figure S15. The optimized geometry of **3d**. Blue – nitrogen, red – oxygen, grey – carbon and off-white – hydrogen atoms. Mulliken charges are provided on atoms.

Cartesian coordinates (Å) for atoms in the optimized geometry of **3d**.

O	0.12066000	-2.35165500	2.03686400
N	-0.59949500	1.05919400	-0.72574000
N	0.74672100	1.03367100	-0.72298500
N	-1.05849100	-1.74096000	1.53779500
N	1.21051900	-1.80550400	1.46310300
N	-2.69013700	0.23569700	-0.18612800
N	2.81757300	0.16495400	-0.20349100
C	-1.31560900	0.14865000	-0.12714300
C	-0.64679500	-0.86561100	0.61322700
C	0.76202000	-0.91673300	0.61300900
C	1.44255900	0.09012500	-0.15446100
C	-3.56401900	-0.82338500	-0.44833700
C	-3.28164000	-2.14400500	-0.75721000
H	-2.26615400	-2.51482000	-0.81982900
C	-4.35079500	-2.98789300	-1.00770400
H	-4.15740700	-4.02689200	-1.24593200
C	-5.66551300	-2.52509400	-0.96449700
H	-6.47927500	-3.21141700	-1.16483500
C	-5.93731100	-1.19687000	-0.68673000
H	-6.95820400	-0.83301700	-0.67882800
C	-4.88011000	-0.33226300	-0.43311200
C	-4.80078100	1.08596300	-0.17573800
C	-5.76037200	2.08133600	-0.03513400
H	-6.81469400	1.84403700	-0.11773900
C	-5.34667100	3.37763200	0.21324600
H	-6.08150600	4.16628100	0.32154100
C	-3.98921300	3.68334100	0.31886600
H	-3.68660700	4.70738500	0.50310700
C	-3.01365200	2.70986900	0.17975400
H	-1.96233300	2.95501900	0.22886000

```
C       -3.43906200    1.41352300   -0.06205700
C        3.70593800   -0.91161100   -0.33154300
C        3.44966000   -2.25511700   -0.55904000
H        2.44427100   -2.64358300   -0.63304400
C        4.53532500   -3.10487300   -0.68966900
H        4.35798100   -4.15988000   -0.86127900
C        5.84487100   -2.63185400   -0.61055600
H        6.67092000   -3.32465500   -0.71625400
C        6.09396700   -1.28540300   -0.41677200
H        7.11042900   -0.91096900   -0.38082300
C        5.01939800   -0.41481800   -0.28419000
C        4.92918100    1.01736800   -0.13906900
C        5.88680400    2.01699100   -0.02048500
H        6.94214000    1.77007000   -0.02949900
C        5.46999700    3.32907700    0.11170500
H        6.20298200    4.12183400    0.20121400
C        4.11092400    3.64290900    0.12337200
H        3.80391400    4.67792800    0.21708000
C        3.13752700    2.66448200    0.00181900
H        2.08803300    2.91697900   -0.02547600
C        3.56444900    1.35101900   -0.12346500
O       -2.11663100   -2.08136900    1.98505400
```

Table S9. The dihedral angles (°) in the optimized geometry of **3d**. The numbering scheme for atoms is analogous to that of the X-ray structure for comparison.

N1 N2 C1 C2 C3 C4	N5 C5..C16	45.8
N1 N2 C1 C2 C3 C4	N6 C17..C28	41.4
C1 N1 N2 C4	C1 C2 C3 C4	4.5
C1 C2 C3 C4	C2 N3 O1 N4 C3 O2	8.4
C2 N3 O1 N4 C3 O2	N1 N2 C1 C2 C3 C4	10.4

The optimized geometry of **4d**.

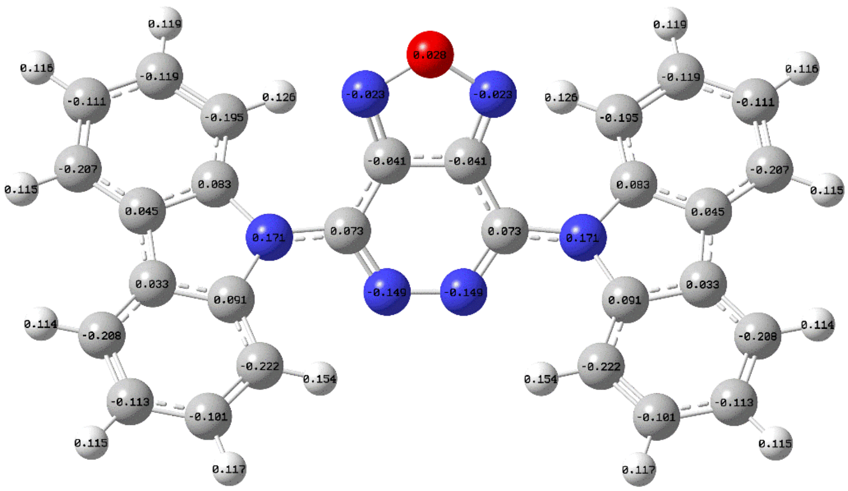

Figure S16. The optimized geometry of **4d**. Blue – nitrogen, red – oxygen, grey – carbon and off-white – hydrogen atoms. Mulliken charges are provided on atoms.

Cartesian coordinates (Å) for atoms in the optimized geometry of **4d**.

O	-0.00010800	-2.51310700	1.91321200
N	-0.67290100	0.91614100	-0.71859900
N	0.67300200	0.91615000	-0.71850600
N	-1.12282200	-1.96013700	1.42862000
N	1.12265800	-1.96019400	1.42867400
N	-2.75834500	0.10718200	-0.16515200
N	2.75837200	0.10714700	-0.16485900
C	-1.38531200	-0.00726900	-0.13580600
C	-0.70840900	-1.03234700	0.60063000
C	0.70832900	-1.03234100	0.60071400
C	1.38533000	-0.00726800	-0.13563700
C	-3.68124500	-0.94244300	-0.27200400
C	-3.47090800	-2.29459300	-0.49545900
H	-2.48010500	-2.71562700	-0.58515000
C	-4.58429200	-3.11099100	-0.60367600
H	-4.44221300	-4.17179500	-0.77223400
C	-5.87711500	-2.59740700	-0.50572300
H	-6.72579700	-3.26487600	-0.59355500
C	-6.08108300	-1.24291500	-0.31558600
H	-7.08466900	-0.83680800	-0.26491900
C	-4.97803600	-0.40540600	-0.20579000
C	-4.84194400	1.02395500	-0.06995300
C	-5.76714200	2.05233800	0.05974400
H	-6.82938600	1.83710600	0.06909100
C	-5.30905900	3.35174600	0.17937900
H	-6.01627000	4.16660200	0.27733600
C	-3.94111900	3.62430900	0.16786200
H	-3.60151600	4.64987500	0.25259700
C	-2.99972300	2.61660200	0.03445000
H	-1.94375800	2.83726200	-0.01043300
C	-3.46770800	1.31598000	-0.07863600

C	3.68126300	-0.94245400	-0.27206400
C	3.47092300	-2.29457500	-0.49570100
H	2.48012200	-2.71561900	-0.58532700
C	4.58430800	-3.11094100	-0.60416700
H	4.44222300	-4.17172300	-0.77286200
C	5.87713500	-2.59735800	-0.50627200
H	6.72581600	-3.26480200	-0.59430000
C	6.08110400	-1.24289400	-0.31594700
H	7.08469100	-0.83678300	-0.26533100
C	4.97806000	-0.40541400	-0.20591000
C	4.84197100	1.02392300	-0.06983300
C	5.76716700	2.05230300	0.05992300
H	6.82941600	1.83708900	0.06910800
C	5.30907500	3.35168300	0.17981200
H	6.01628200	4.16653600	0.27781800
C	3.94112700	3.62422700	0.16849700
H	3.60151900	4.64977400	0.25344400
C	2.99973700	2.61652500	0.03503800
H	1.94376100	2.83717500	-0.00966500
C	3.46773300	1.31593100	-0.07831400

Table S10. The dihedral angles (°) in the optimized geometry of **4d**. The numbering scheme for atoms is analogous to that of the X-ray structure for comparison.

N1 N2 C1 C2 C3 C4	N5 C5..C16	47.0
N1 N2 C1 C2 C3 C4	N6 C17..C28	47.0
C2 N3 O1 N4 C3	N1 N2 C1 C2 C3 C4	5.5
C2 N3 O1 N4 C3	C1 C2 C3 C4	6.7
C1 C2 C3 C4	N1 N2 C1 C4	5.1

Location of frontier orbitals.

In both cases **3d** and **4d**, HOMO is localized at entire π-system with the exception of the furoxan/furazan ring (see Figure S17 below). The LUMO for both cases is localized at the central $N_4C_4O_2$/N_4C_4O heterocycle and partly at the nitrogen atom of each N-carbazolyl group, but not at carbon atoms of the latter.

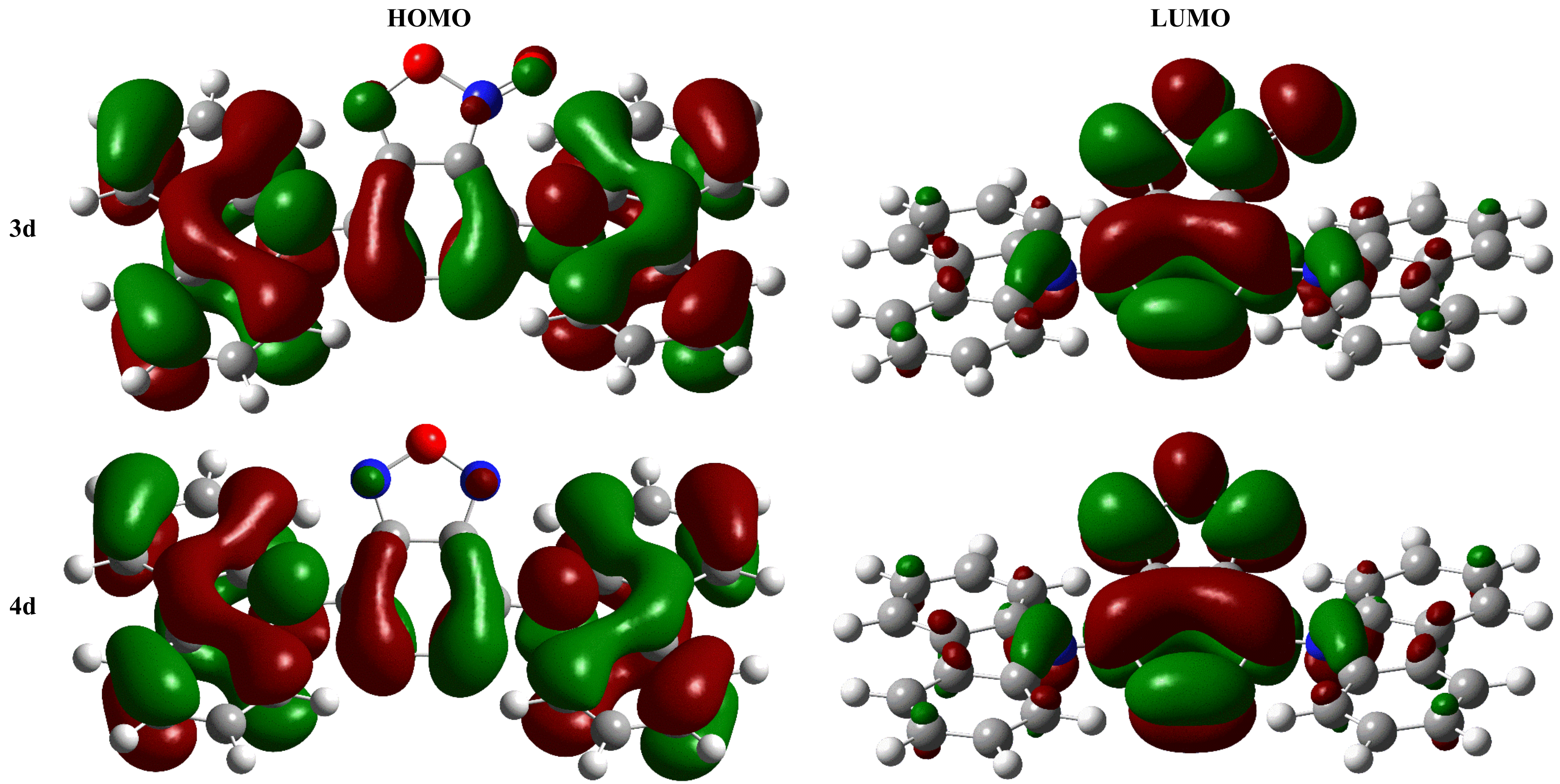

Figure S17. The optimized geometry of **4d**. Blue – nitrogen, red – oxygen, grey – carbon and off-white – hydrogen atoms. Mulliken charges are provided on atoms.

5. References

1. CrysAlisPro. *Rigaku Oxford Diffraction*, version 1.171.42.89a, **2023** for **3d** and version 1.171.41.106a, **2021** for **4d**.
2. Sheldrick, G.M. SHELXT - Integrated space-group and crystal-structure determination. *Acta Cryst.* **2015**, A71(1), 3-8. https://doi.org/10.1107/S2053273314026370
3. Sheldrick, G.M. Crystal structure refinement with SHELXL. *Acta Cryst.* **2015**, C71(1), 3-8. https://doi.org/10.1107/S2053229614024218
4. Dolomanov O.V.; Bourhis L.J.; Gildea R.J.; Howard J.A.K.; Puschmann H. OLEX2: a complete structure solution, refinement and analysis program. *J. Appl. Cryst.* **2009**, 42(2), 229-341. https://doi.org/10.1107/S0021889808042726
5. Spackman, P.R.; Turner, M.J., McKinnon, J.J.; Wolff, S.K.; Grimwood, D.J.; Jayatilaka, D.; Spackman, M.A. *CrystalExplorer*: a program for Hirshfeld surface analysis, visualization and quantitative analysis of molecular crystals. *J. Appl. Cryst.* **2021**, 54(3), 1006-1011. https://doi.org/10.1107/S1600576721002910
6. Sheldrick, G.M. A short history of SHELX. *Acta Cryst.,* **2008**, A64(1), 112-122. http://dx.doi.org/10.1107/S0108767307043930
7. Macrae, C. F.; Sovago, I.; Cottrell, S. J.; Galek, P. T. A.; McCabe, P.; Pidcock, E.; Platings, M.; Shields, G. P.; Stevens, J. S.; Towler, M.; Wood, P. A. Mercury 4.0: from visualization to analysis, design and prediction. *J. Appl. Cryst.* **2020**, 53(1), 226-235. https://doi.org/10.1107/S1600576719014092
8. C. Adamo, V. Barone. Toward reliable density functional methods without adjustable parameters: The PBE0 model. *J. Chem. Phys*. **1999**, *110(13)*, 6158–6170. DOI: https://doi.org/10.1063/1.478522
9. F. Weigend. Accurate Coulomb-fitting basis sets for H to Rn. *Phys. Chem. Chem. Phys*. **2006**, *8(9)*, 1057-1065. https://doi.org/10.1039/B515623H
10. Gaussian 16, Revision C.01, M. J. Frisch, G. W. Trucks, H. B. Schlegel, G. E. Scuseria, M. A. Robb, J. R. Cheeseman, G. Scalmani, V. Barone, G. A. Petersson, H. Nakatsuji, X. Li, M. Caricato, A. V. Marenich, J. Bloino, B. G. Janesko, R. Gomperts, B. Mennucci, H. P. Hratchian, J. V. Ortiz, A. F. Izmaylov, J. L. Sonnenberg, D. Williams-Young, F. Ding, F. Lipparini, F. Egidi, J. Goings, B. Peng, A. Petrone, T. Henderson, D. Ranasinghe, V. G. Zakrzewski, J. Gao, N. Rega, G. Zheng, W. Liang, M. Hada, M. Ehara, K. Toyota, R. Fukuda, J. Hasegawa, M. Ishida, T. Nakajima, Y. Honda, O. Kitao, H. Nakai, T. Vreven, K. Throssell, J. A. Montgomery, Jr., J. E. Peralta, F. Ogliaro, M. J. Bearpark, J. J. Heyd, E. N. Brothers, K. N. Kudin, V. N. Staroverov, T. A. Keith, R. Kobayashi, J. Normand, K. Raghavachari, A. P. Rendell, J. C. Burant, S. S. Iyengar, J. Tomasi, M. Cossi, J. M. Millam, M. Klene, C. Adamo, R. Cammi, J. W. Ochterski, R. L. Martin, K. Morokuma, O. Farkas, J. B. Foresman, and D. J. Fox, Gaussian, Inc., Wallingford CT, **2016**.
